# Short‐Chained Anthracene Strapped Porphyrins and their Endoperoxides†

**DOI:** 10.1002/ejoc.202000283

**Published:** 2020-04-20

**Authors:** Susan Callaghan, Keith J. Flanagan, John E. O'Brien, Mathias O. Senge

**Affiliations:** ^1^ School of Chemistry Trinity College Dublin The University of Dublin Trinity Biomedical Sciences Institute 152‐160 Pearse Street Dublin 2 Ireland; ^2^ Institute for Advanced Study (TUM‐IAS) Technische Universität München Lichtenberg‐Str. 2a 85748 Garching Germany

**Keywords:** Strapped Porphyrins, Endoperoxide, Singlet oxygen, Conformational analysis, Porphyrinoids

## Abstract

The syntheses of short‐chained anthracene‐strapped porphyrins and their Zn(II)complexes are reported. The key synthetic step is a [2+2] condensation between a dipyrromethane and an anthracene bisaldehyde, 2,2'‐((anthracene‐9,10‐diylbis(methylene))bis(oxy))dibenzaldehyde. Following exposure to white light, self‐sensitized singlet oxygen and the anthracene moieties underwent [4+2] cycloaddition reactions to yield the corresponding endoperoxides. ^1^H NMR studies demonstrate that the endoperoxide readily formed in [D]chloroform and decayed at 85 °C. X‐ray crystallography and absorption spectroscopy were used to confirm macrocyclic distortion in the parent strapped porphyrins and endoperoxides. Additionally, X‐ray crystallography indicated that endoperoxide formation occurred exclusively on the outside face of the anthracene moiety.

## Graphical abstract

Herein, we report the synthesis of three short‐chained anthracene strapped porphyrins and their corresponding endoperoxides, formed by a cycloaddition reaction between in situ generated singlet oxygen and the anthracene moiety. We also synthesized Zn(II)complexes of the strapped porphyrins. X‐ray crystallography and UV/Vis analysis confirmed macrocyclic distortion in the strapped systems.

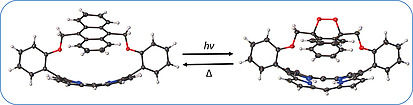

## Introduction

Endoperoxide formation, mediated by [4+2] cycloaddition reactions between an aromatic unit and singlet oxygen, is an emerging strategy for modulating the photochemistry of singlet oxygen. Since this reaction was first described by Moureu, Dufraisse, and Dean,^1^ it has found application in bioimaging and is the underlying interaction in probes, for example, singlet oxygen sensor green and a fluorescence probe designed by Mokhir and co‐workers.^2^ Additionally, endoperoxides of moieties including anthracene, pyridone, and naphthalene have been shown to thermally decay to slowly release singlet oxygen. These systems have found application in therapeutics,^3^ oxygen storage devices,^4^ and even photolithography.^5^

Porphyrin macrocycles can take on non‐planar conformations given the correct conditions. A common method to introduce macrocycle distortion is the substitution of the periphery or core with bulky groups.^6^ These distortion properties are also endowed by short strapped systems, i.e. porphyrins with a connection between two meso‐meso or β‐β carbon atoms, and the nature of the induced distortion can affect the shape and size of the cavity.^7^ Strapped systems have been extensively studied, especially as possible heme mimics as the porphyrin cores in these active sites are distorted from planarity due to surrounding proteins.^8^ In addition, macrocyclic distortion can lead to interesting chemical properties including organocatalytic activity and use in sensing applications.^6^, [7a], 9 Early systems that achieved significant distortion using short alkyl chains were prepared in the 1980s by the groups of Dolphin, Einstein, and Walker.^10^ One may also recall the pioneering works of Staab on porphyrin quinone cyclophanes.^11^

**Figure 1:**
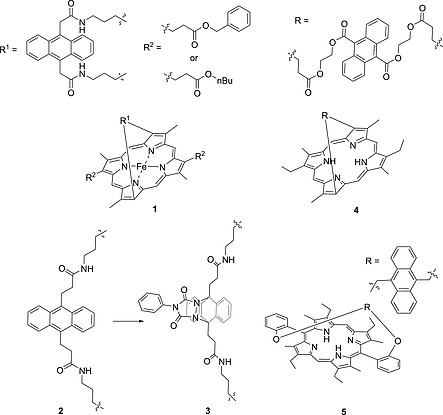
Examples of previously reported anthracene strapped porphyrins.

Previous reports of anthracene containing strapped systems (Figure 1, porphyrins** 1**–**4**) did not describe macrocyclic distortion.^12^ In one study, Traylor and co‐workers synthesized anthracene strapped porphyrins as part of a wider study related to ligand binding in natural heme proteins. The anthracene unit was introduced in a double amide bond‐forming reaction between an anthracene containing acid chloride unit and an amine‐containing porphyrin. To further restrict the binding pocket, they performed a cycloaddition reaction with 1‐phenyl‐1H‐pyrrole‐2,5‐dione to yield compound **3**. To the best of our knowledge, this is the only example of a cycloaddition reaction across an anthracene strapped porphyrin.[12b] In 1991, Osuka et al. reported porphyrin **5** and were able to confirm that strap length was related to macrocyclic distortion using NMR and absorption spectroscopy.^13^ Moreover, recent interest in the development of general methods for the synthesis of strapped porphyrin systems and their stereochemical properties has revitalized interest in this family of tetrapyrroles.^14^

With these concepts in mind, we designed anthracene strapped systems with a short chain to induce macrocyclic distortion and restrict the size of the cavity, thereby shielding one face of the anthracene moiety. The anthracene moiety acts as the site for a reversible [4+2] cycloaddition with self‐sensitized singlet oxygen via action of the porphyrin photosensitizer component. The reversibility at elevated temperatures is expected to find application in oxygen storage devices.

## Results and Discussion

**Scheme 1:**
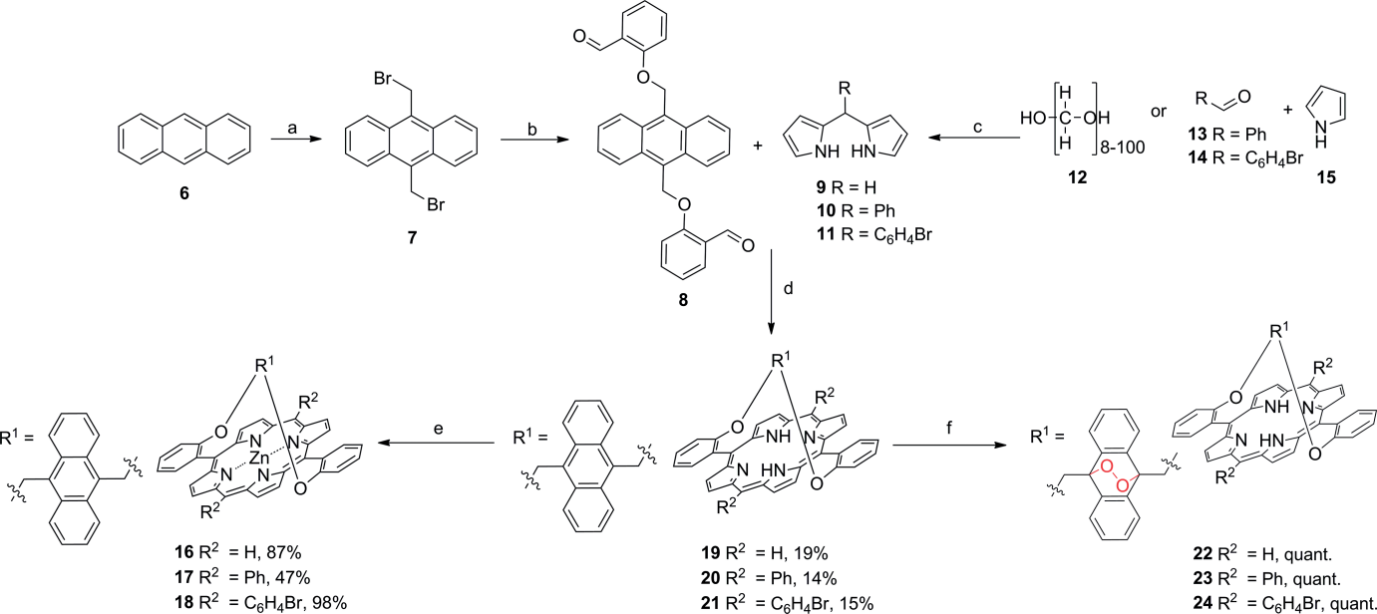
Synthesis of parent porphyrins **19–21**, endoperoxides **22**–**24**, and Zn(II) complexes **16**–**18**. a) paraformaldehyde, 33 % HBr in acetic acid, AlCl_3_, 3 h, 50 °C; b) salicylaldehyde, K_2_CO_3_, DMF, KI, 24 h, 60 °C; c) TFA (cat.), 30 min, r.t.; d) 1. DCM 2. TFA, 3.5 h, r.t. 3. TEA, *p*‐chloranil, 70 °C, 1 h; e) Zn(II)acetate, methanol, DCM, 80 °C, 12 h; f) [D]chloroform, *hv*, 15 min.

Single strapped porphyrins (Scheme 1, porphyrins** 19**–**21**) were prepared using condensation reactions between an anthracene containing bisaldehyde, 2,2'‐((anthracene‐9,10‐diylbis(methylene))bis(oxy))dibenzaldehyde **8**, and dipyrromethane (DPM) or a DPM derivative. This approach was chosen to ensure that the “*trans*” (5,15) isomer would be the major product as we aimed to position the anthracene moiety above the porphyrin core to induce maximum macrocycle distortion. This would also differentiate the two faces of the anthracene unit with regard to endoperoxide formation. Firstly, bromine was introduced to unsubstituted anthracene (compound **6**) using paraformaldehyde, 33 % HBr in acetic acid and AlCl_3_. A substitution reaction with salicylaldehyde in the presence of a base and KI in DMF yielded the bisaldehyde **8**, using a procedure adapted from literature.^15^ Both reactions produced high yields (up to 97 % for compound **6** and 60 % for compound **7**), which were suitable for multigram scale synthesis.

We then synthesized DPM **9** using a literature adapted procedure and prepared the DPM derivatives **10** and **11** to allow for the expansion of the porphyrin library (Scheme 1).^16^ Either paraformaldyehdye, benzaldehyde or bromobenzaldehyde was condensed with excess pyrrole using trifluoroacetic acid (TFA) as a catalyst. The reactions were monitored using a bromine chamber. Yields between 28 % and 79 % were obtained and these condensation reactions were performed on multigram scales.

Each DPM (**9**–**11**) was then condensed with the bisaldehyde **8**, using dichloromethane (DCM) and TFA as an acid catalyst. After 3.5 h at r.t., triethylamine (TEA) was added to neutralize the TFA (Scheme 1). An oxidation step using *p*‐chloranil followed. This step is key to inducing macrocyclic distortion. As the initially formed porphyrinogen is not conjugated the structure is not rigid. Upon oxidation, the macrocycle is flattened and a “bowstring effect” induces distortion. The porphyrins were purified using column chromatography and recrystallization and yields between 14 % and 19 % were obtained (Scheme 1). A similar synthetic strategy was employed by Osuka et al. for the synthesis of porphyrin** 5** and they obtained a 25 % yield, which is consistent with our moderate yields.^13^

We also synthesized the Zn(II)metallated derivatives of porphyrins **19**–**21** to yield porphyrins **16**–**18**. This was achieved using Zn(II)acetate, methanol and DCM as solvents and yields between 47 % and 98 % were obtained (Scheme 1). It was found that heating to 80 °C compared to initial attempts at r.t., decreased the reaction time. The products were purified using silica chromatography and recrystallization.

**Figure 2:**
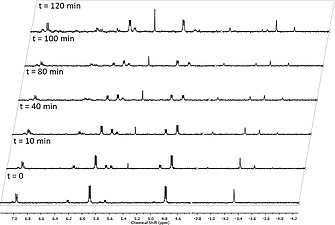
Time evolution of ^1^H NMR spectra of endoperoxide **19** in [D_6_]DMSO at 85 °C over 2 h.

Endoperoxides of the parent porphyrins (**22–24**) were then prepared. The parent porphyrins were dissolved in [D]chloroform and irradiated with a white light source in an NMR tube. The reactions were monitored by ^1^H NMR and yields were quantitative in all cases. We then used endoperoxide **22** to study thermal decay. After endoperoxide formation, the [D]chloroform was removed and replaced with deuterated [D_6_]DMSO. The sample was heated to 85 °C in accordance with other anthracene derivatives to induce thermal decay of the endoperoxide.^17^ At *t* = 0 h the NH proton signal of endoperoxide **22** is observed at –3.34 ppm. In the aromatic region, we observed the CH protons of the anthracene endoperoxide as two multiplets at 5.91 and 4.81 ppm. It is noteworthy that the CH protons of the anthracene endoperoxide have a lower chemical shift than unsubstituted anthracene, which is attributed to ring current effects. Following heating over 2 h, the signals for the CH protons of the anthracene endoperoxide at 5.91 and 4.81 ppm slowly decay with an appearance of aromatic signals at 5.74 and 4.97 ppm that correspond to the CH protons of anthracene. Only minor changes in the chemical shifts of the CH protons of the anthracene and endoperoxide units are noted as we expect they are under the influence of similar ring current effects. The NH protons also undergo changes. The endoperoxide signal at –3.34 ppm decays and the parent NH signal appears at –3.50 ppm. The endoperoxide NH signal is deshielded in comparison to the parent because of the electronegative oxygen atoms of the endoperoxide. Over the course of the 2 h we also see an emergence of other signals, which are especially evident in the NH region. These signals may indicate the formation of rearranged endoperoxide porphyrin products; thus, it can be concluded that the thermal decay of **19** in [D_6_]DMSO is not quantitative (Figure 2).^17^

We attempted the synthesis of the analogous “*trans*” (5,15 and 10,20) double‐strapped system of porphyrin **19** to further increase macrocyclic distortion. Firstly, we adopted the direct synthesis approach, which was used by Reddy and Chandrashekar^18^ in the synthesis of similar phenyl‐strapped porphyrins. We condensed pyrrole with compound **8** under both Adler Longo and Lindsey conditions^19^ on multigram scales. In a second approach, we used compound **8** to synthesize the corresponding bisDPM and then condensed this in a 1:1 ratio with compound **8** under Lindsey conditions. The product was identified by mass spectrometry (HRMS (MALDI‐TOF) *m/z* calcd. for C_70_H_51_N_4_O_4_ [M + H]^+^: 1083.3910, 1083.3909 found) but pure isolation was not achieved due to π‐stacking of the anthracene moieties.

**Figure 3:**
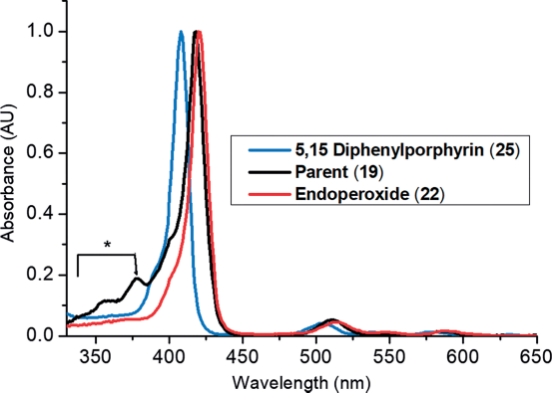
Normalized absorption spectra of porphyrins **25**,** 19**, and** 22** in DCM as an example to show how the strap induces distortion. *Absorption wavelength range for anthracene moiety.

To further investigate changes in the macrocycle core upon endoperoxide formation and the introduction of a short strap we studied the absorption spectra of the strapped systems and compared them to 5,15‐diphenylporphyrin **25** 20 as a non‐strapped analogue. In Figure 3, the normalized absorption spectra of 5,15‐diphenylporphyrin **25** (blue), parent porphyrin **19** (black), and endoperoxide, **22** (red) recorded in DCM are presented to exemplify this phenomenon. We can see that 5,15‐ diphenylporphyrin (**25**) has a Soret absorption maximum at 408 nm and upon the introduction of the strap, there is a red‐shift of 10 nm to 418 nm (**19**), an indication of increased macrocycle distortion.^21^ The slight bathochromic shift of 2 nm between the parent porphyrin **19** and endoperoxide **22** may indicate minor changes in macrocyclic distortion. Also of note are the bands between 330–400 nm in **19** that represent the anthracene moiety. These are not present in the endoperoxide absorption spectrum as conjugation is disrupted. The red‐shift pattern is also repeated for porphyrins **20**, **21**, **23**, and **24**, which show a significant bathochromic shift when compared to 5,15‐diphenylporphyrin (**25**) and a minor shift upon endoperoxide formation (Table 1).

**Table 1:** Absorption maxima (*λ*_max_) of strapped porphyrins, endoperoxides and 5,15‐diphenylporphyrin, **25** recorded in DCM

Porphyrin	*λ*_max_ [nm]
**25**	408, 504, 538, 577, 632
**19**	359, 378, 418, 511, 543, 584, 632
**22**	420, 515, 545, 587, 632
**20**	360, 384, 431, 526, 564, 600, 658
**23**	433, 528, 567, 601, 657
**21**	361, 382, 432, 527, 565, 601, 658
**24**	434, 529, 566, 604, 660

**Figure 4:**
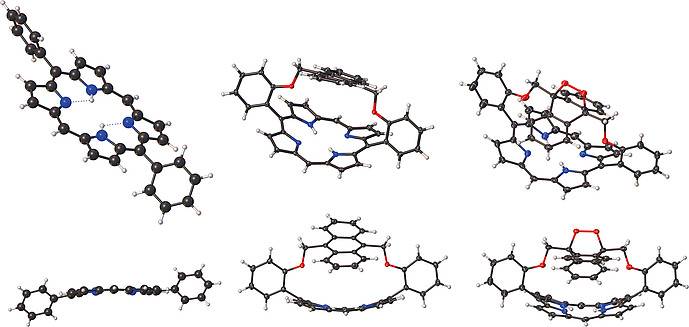
Molecular structure in the crystal of **25**,^20^ **19**, and **22** (left to right) viewed at a tilted angle (top) and the side view (bottom). Thermal displacements are given at 50 % for **19** and **22**. The structure of **25** is drawn isotropically.

In order to investigate the macrocycle conformation in more detail, single‐crystal X‐ray crystallographic studies were undertaken and the X‐ray crystal structures of porphyrins **19** and **22** were determined (Figure 4). The crystal structure of **22** showed endoperoxide formation exclusively on the outer face of the anthracene moiety, thus making this a face‐selective photoreaction. ^1^H NMR spectroscopy did not provide any evidence of inner face endoperoxide formation in solution as only one NH signal was observed for the endoperoxide. If the endoperoxide oxygens were on the inner face of the anthracene directly above the porphyrin plane we would expect a different NH chemical shift to that found for structure **22**. We postulate that this is a result of steric hindrance as the outer face is more available for binding and repulsion form the electronegative cavity.

To study the relative effects of the strap and endoperoxide formation on macrocyclic distortion the structures of **19** and **22** were compared to that of 5,15‐diphenylporphyrin **25**. Two structures have been reported in the literature for the latter; a DCM solvated form (**25**‐DCM) and one without solvent molecules (**25**).^22^ Figure 4 shows that there is significant macrocycle distortion in porphyrins **19** and** 22**, which contain straps, compared to 5,15‐diphenylporphryin, **25**. The structural differences are quite drastic and have been graphically outlined in Fig. S1–S8 with different views of both porphyrins **19** and** 22**.

The skeletal deviation plots show that the strapped systems (porphyrins **19** and **22**) display increased out‐of‐plane ring distortion, with the meso‐carbons presenting the largest deviations (Fig. S9). In the normal‐coordinate structural decomposition (NSD)^23^, ^24^, ^25^ plots – a means to identify and quantify macrocycle distortion modes – a simple trend becomes evident. Porphyrins **19** and **22** show an inverse relationship between out‐of‐plane (*oop*) and in‐plane (*ip*) distortion modes. This is represented by an increase in the *oop* modes while the *ip* modes are significantly decreased (Fig. S10). Both **25** and **25**‐DCM show a preference for the ruffled (*B*_1*u*_) mode with **25**‐DCM having a slightly increased contribution to this mode. For porphyrins **19** and **22** there is an increase in the contribution of the *B*_1*u*_ mode with a second smaller contribution to the domed (*A*_2*u*_) mode. The structure of **22** shows a slightly larger contribution to the *B*_1*u*_ mode compared to **19** which is also reflected in the Δ*_oop_* with the evident trend being **25** ≤ **25**‐DCM < **19** ≤ **22**. Moving to the *ip* distortion modes both **25** and **25**‐DCM have significant contributions to the meso‐stretching (*B*_2*g*_) mode with a secondary contribution to the breathing (*A*_1*g*_) mode. In the structure of **19**, a decrease in the *B*_2*g*_ mode is evident compared to **25** with little to no contribution noted in the other *ip* distortion modes. For the structure of compound **22**, the main contributions have now shifted to the N‐stretching (*B*_1*g*_) and *A*_1*g*_ modes with almost equal contributions to both modes. The specific trend seen in the Δ*_ip_* is **25** ≤ **25**‐DCM > **19** ≥ **22**.

The geometrical changes in the porphyrin macrocycle are listed in Table 2. The bond lengths around the porphyrin macrocycle do not show any significant changes. A similar situation is noted in the bond angles; however, in four areas there are significant changes to be noted. These are in the N–C_α__(1,9,11,19)_–C_m(10,20)_, C_α__(4,14)_–C_m(5,15)_–C_α(6,16)_, C_α__(1,11)_–C_m(10,20)_–C_α__(9,19)_, and C_m(10,20)_–C_α__(1,9,11,19)_–C_β__(2,8,12,18)_ angles of the porphyrin macrocycle. The first thing to note is the effect the inclusion of DCM molecule has on **25** and **25**‐DCM. There is a 0.7° difference between the C_α__(1,11)_–C_m(10,20)_–C_α__(9,19)_ angle. However, there are only minor deviations between the other angles. This suggests that the inclusion of the solvent molecule does not greatly affect the overall structure. Moving to the structure of **19** compared to **25**, the N–C_α__(1,9,11,19)_–C_m(10,20)_, C_α__(4,14)_–C_m(5,15)_–C_α__(6,16)_, and C_m(10,20)_–C_α__(1,9,11,19)_–C_β(2,8,12,18)_ angles increase by 2°, 2.5°, and 1.8°, respectively. Conversely, the C_α__(1,11)_–C_m(10,20)_–C_α__(9,19)_ angle is reduced by 3°. A small change of 0.9° is noted in the C_m(5,15)_–C_α__(4,6,14,16)_–C_β__(3,7,13,17)_ and N–C_α__(4,6,14,16)_–C_m(5,15)_ angles. A similar change is noted in the structure of **22**; however, the C_α__(4,14)_–C_m(5,15)_–C_α__(6,16)_ angle shows a 1.3° reduction compared to **19**. All other angles only deviate by at most 0.5° between **22** and **19**, suggesting that endoperoxide formation only has minor effects on the porphyrin macrocycle, which is evident in both the NSD and skeletal deviation plots (Fig. S9 and S10) and is consistent with the UV/Vis data presented in Table 1.

**Table 2:** Averaged geometrical parameters for bond lengths, angles, core conformation, and atom displacements of **25**, **25**‐DCM, **19**, and **22**

	**25l** 22	**25‐**DCM^22^	**19**	**22**
Bond lengths [Å]				
N–C_α_	1.368(15)	1.367(16)	1.371(4)	1.368(11)
C_α_–C_β_	1.440(17)	1.442(16)	1.440(4)	1.438(11)
C_α_‐Cm(5,15)	1.408(17)	1.401(18)	1.401(4)	1.403(10)
C_α_‐Cm(10,20)	1.388(16)	1.389(14)	1.390(4)	1.390(12)
C_β_–C_β_	1.357(17)	1.355(14)	1.354(5)	1.353(11)
*Bond angles (°)*				
N–C_α_(_4,6,14,16_)–C_m_(_5,15_)	124.5(11)	124.8(11)	125.4(13)	124.9(4)
N–C_α(1,9,11,19)–_C_m(10,20)_	127.1(11)	126.9(12)	125.1(9)	125.4(4)
N–C_α(4,6,14,16)–_C_β (3,7,13,17)_	108.7(10)	108.8(11)	108.8(8)	108.8(5)
N–C_α(1,9,11,19)_–C_β (2,8,12,18)_	109.1(10)	108.9(11)	108.9(8)	108.7(7)
C_α_–N–C_α_	107.7(10)	107.8(11)	107.6(3)	107.7(5)
C_α(4,14)_–C_m(5,15)_–C_α(6,16)_	122.7(10)	123.3(12)	125.2(12)	123.9(4)
C_α(1,11)_–C_m(10,20)_–C_α(9,19)_	129.1(12)	128.4(12)	126.1(11)	126.4(5)
C_α4,6,14,16)_–C_β(3,7,13,17)_–C_β(2,8,12,18)_	107.3(10)	107.1(12)	107.4(13)	107.2(6)
C_α(1,9,11,19)_–C_β(2,8,12,18)_–C_b(3,7,13,17)_	107.2(11)	107.4(12)	107.3(13)	107.4(5)
C_m(5,15)_–C_α(4,6,14,16)_–C_β(3,7,13,17)_	126.7(10)	126.4(12)	125.8(11)	126.3(5)
C_m(10,20)_–C_α(1,9,11,19)_–C_β(2,8,12,18)_	123.8(12)	124.1(12)	125.6(11)	125.2(5)
Pyrrole tilt (°)				
*N21*	3.6(4)	5.2(4)	14.5(5)	16.9(14)
*N22*	4.5(4)	5.1(4)	15.0(4)	17.3(2)
*N23*	5.3(4)	5.7(4)	14.8(7)	18.1(17)
N24	5.9(4)	4.8(4)	13.8(6)	16.0(17)
Structural parameters [Å]				
C_ortho_ **···**C_ortho_ a	11.580(17)	11.506(2)	8.967(2)	8.928(9)
Δ *_ip_* b	0.450	0.383	0.114	0.105
Δ *_oop_* c	0.429	0.462	1.261	1.496
N21**···**N22^d^	2.757(15)	2.772(15)	2.882(2)	2.823(3)
N22**···**N23^d^	3.065(13)	3.238(15)	2.892(3)	2.912(3)
N23**···**N24^d^	2.750(15)	2.775(15)	2.886(2)	2.817(3)
N24**···**N21^d^	3.055(13)	3.031(15)	2.853(3)	2.893(3)
Δ *24* e	0.091	0.095	0.258	0.306
Δ *N* f	0.024	0.025	0.159	0.159
Δ *C_m_*_(5,15)_ g	0.142	0.162	0.425	0.505
Δ *C_m_*_(10,20)_ g	0.136	0.153	0.395	0.482
ΔC_α_ h	0.082	0.092	0.238	0.286
Δ *C_β_* i	0.064	0.065	0.157	0.201

a Calculated distance between the C_ortho_ carbon atoms to simulate the length of the strap between the 5,15‐substituted phenyl ring.

b Simulated total in‐plane distortion.

c Simulated total out‐of‐plane distortion.

d Calculated distance between the pyrrole nitrogen atoms.

e Average deviation from the least‐squares plane of the 24‐macrocycle atoms.

f Simulated displacement of the four internal nitrogen atoms from the 24‐atom mean plane.

g Deviation of the meso‐carbon atoms from the 24‐atom mean plane.

h Average deviation of the α‐carbon atoms from the 24‐atom mean plane.

i Average deviation of the β‐carbon atoms from the 24‐atom mean plane.

Looking at the pyrrole tilt angles a specific trend becomes apparent. For the N21, N22, and N23 pyrrole rings the tilt angle trend is **25** ≤ **25**‐DCM < **19** ≤ **22** with an average 0.4–1.6° increase from **25** to **25**‐DCM, a ca. 9° increase from **25**‐DCM to **19**, and a 2.4–3.2° increase from **19** to **22**. The only deviation from this trend is seen in N24 where the trend becomes **25**‐DCM ≤ **25** < **19** ≤ **22**. Moving to the atom deviations the **25** ≤ **25**‐DCM < **19** ≤ **22** is followed for the Δ*24*, Δ*N*, Δ*C_m_*_,_ Δ*C*_α_, and Δ*C_β_* deviations from the 24‐atom least‐squares‐plane. These changes are quite evident in the NSD where there is a significant increase in the *oop* distortion modes, as demonstrated by the increase in the Δ*_oop_* following the addition of the 5,15‐strap.

The final structural parameter to look at is how the size of the core (N_x_ **···**N_x_) changes between the porphyrins. The most obvious change is moving from the “rectangular” shape where the 5,15‐axis is longer than the 10,20‐axis, to a “squarer” shape where both the 5,15‐ and 10,20‐axes are approximately the same distance. The former core elongation is often encountered in 5,15‐disubstituted porphyrins, while the latter is typical for symmetric A_4_‐type porphyrins.^6^, ^26^ This is also affected by the length of the strap (as calculated from the distance between the C_ortho_ **···**C_ortho_ of the 5,15‐phenyl substituents). Without a strap, the distance between these two atoms is in the range of 11.506–11.580 Å. Upon the addition of the strap, this distance is significantly shortened to 8.967(2) Å (**19**) with a smaller decrease in distance to 8.928(9) Å following endoperoxide formation (**22**). This is reflected in the *ip* distortion modes with a significant decrease in the Δ_*ip*_ following the addition of the 5,15‐strap, which prevents core elongation. From the above observations, it is clear that while there are only minor changes to the bond lengths and angles in the macrocycle ring the pyrrole tilts, atom deviations, and core elongation are changed as a result of the introduction of a strap between the 5,15‐meso‐substituents.

**Figure 5:**
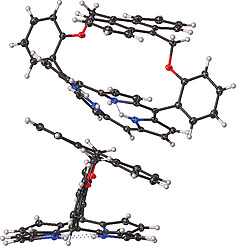
Stacking interactions seen between moieties of **19**.

**Figure 6:**
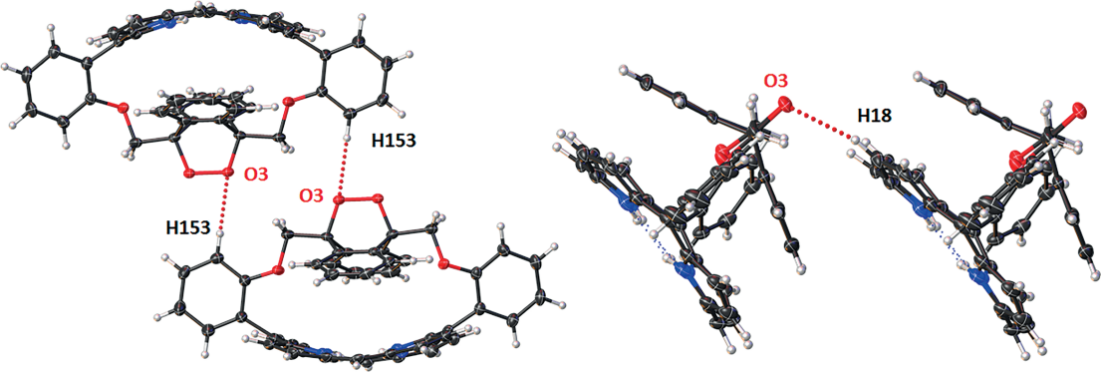
Stacking interactions seen in 22 showing the head‐to‐head interaction [C–H153**···**O3 (2.479(2) Å, 168.7(2)°)] (left) and the edge‐on interaction [C–H18**···**O3 (2.620(2) Å, 152.7(2)°)] (right).

Finally, there are several structural changes in the packing patterns (Fig. S11–S14). In the structure of **25**, there is evidence of stacking interactions between the porphyrin rings (Fig. S15). However, there is no indication of π‐stacking between the porphyrin layers. When a solvent DCM molecule is included in the stacking, the pattern is skewed to form a tilted edge‐on interaction between the porphyrin layers (Fig. S16). With the introduction of the strap across the 5,15‐meso‐substituents, several changes occur; the first is to note that the spacing between the porphyrin macrocycles is now expanded due to the strap creating a buffer zone between molecules as seen in **19** (Figure 5). No specific interactions are noted in the structure of **19**. However, in the structure of **22**, there is a clear head‐to‐head interaction between the endoperoxide moiety and the meso‐substituent [C–H153**···**O3 (2.479(2) Å, 168.7(2)°)], which is directive in the crystal packing (Figure 6, left). This is accompanied by an edge‐on interaction between the porphyrin macrocycle and the endoperoxide moiety [C–H18**···**O3 (2.620(2) Å, 152.7(2)°)] (Figure 6, right). As seen in the crystal packing, this results in head‐to‐head interactions that give rise to the loose packing system of porphyrin **25**.

## Conclusions

In summary, we presented the synthesis of three short‐chained anthracene strapped porphyrins, their corresponding endoperoxides and Zn(II)complexes. The porphyrins were accessed using [2+2] condensation reactions, and endoperoxide formation was achieved with white light in [D]chloroform selectively on the outside of the anthracene strap. Upon heating to 85 °C, endoperoxide decay was observed and monitored by ^1^H NMR. As seen in the X‐ray structures of **19** and **22**, endoperoxide formation caused bending of the anthracene moiety to flank the core. This interesting structural effect has the potential for reversible on‐off porphyrin core shielding and chemical modification of the strap may lead to a fully inaccessible core on one side. In the future, this approach may be utilized for switchable selective sensing applications. The X‐ray crystal structures of a parent porphyrin and its corresponding endoperoxide were presented and macrocyclic distortion was confirmed with an *oop* distortion of 1.261 Å for the parent and 1.496 Å for the endoperoxide, which can be compared to 0.429–0.462 Å for 5,15‐diphenylporphyrin, **25**. For the same porphyrins, a 10–12 nm red‐shift was observed in the UV/Vis spectra compared to 5,15‐diphenylporphyrin, **25**, further confirming the macrocyclic distortion in solution.

## Experimental Section

X‐ray Crystallography: The crystals were grown following the protocol developed by Hope by dissolving the porphyrins in either DCM or chloroform and layering with a second solvent (methanol or hexane) for liquid diffusion or allowing for slow evaporate over time.^27^ Single crystal X‐ray diffraction data for all porphyrins were collected on a Bruker APEX 2 DUO CCD diffractometer by using graphite‐monochromated Mo *K*_α_ (*λ* = 0.71073 Å) radiation. The crystals were mounted on a MiTeGen MicroMount and collected at 100(2) K by using an Oxford Cryosystems Cobra low‐temperature device. The data were collected by using omega and phi scans and were corrected for Lorentz and polarization effects by using the APEX software suite.^28^, ^29^, ^30^ Using Olex2, the structure was solved with the XT structure solution program, using the intrinsic phasing solution method and refined against |*F*_2_| with XL using **least‐squares** minimization.^31^, ^32^ Hydrogen atoms were generally placed in geometrically calculated positions and refined using a riding model. The crystal data and details of data refinements can be found in Table S1. All images were prepared by using Olex2.^31^ The carbon and nitrogen bound hydrogen atoms were placed in their expected calculated positions and refined as riding model: N–H = 0.88 Å, C–H = 0.95–0.98 Å, with 1.2 Ueq (C, N) for all hydrogen atoms. In the structure of **19**, the distances between H36B**···**H31 and H34**···**H21A were fixed to remove the short contact present in the structure. The inner core nitrogen atoms were modelled over two positions at an occupancy of 50:50 %. In the structure of **22**, the inner core nitrogen atoms were modelled over two positions at an occupancy of 50:50 %.

**Normal‐coordinate Structural Decomposition (NSD) Analysis:** The theoretical background and development of this method were described by Shelnutt and co‐workers.^23^, ^24^, ^25^ NSD is a conceptually simple method that employs the decomposition of the conformation of the macrocycle by a basis set composed of its various normal modes of vibration,^33^ affording clear separation of the contributing distortions to the macrocycle conformation in a quantitative fashion. For calculations, we used the NSD engine program as provided by Shelnutt.^34^

**Synthetic and Analytical Methods**. All chemicals were commercially sourced and used without further purification. Dry DCM was obtained by passing through alumina under N_2_ in a solvent purification system and then further dried with activated molecular sieves. Analytical thin‐layer chromatography was performed using silica gel 60 (fluorescence indicator F254, pre‐coated sheets, 0.2 mm thick, 20 cm × 20 cm; Merck) plates and visualized by UV irradiation (*λ* = 254 nm). Column chromatography was carried out using Fluka Silica Gel 60 (230–400 mesh; Merck). UV/Vis spectra were recorded in solutions using a Specord 250 spectrophotometer from Analytic Jena (1 cm path length quartz cell). Photo‐irradiations were performed in an NMR tube using a white light source (Philips, 15V–150 W lamp), equipped with a 400 nm cut‐off filter (Schott GG 400). The NMR spectra were recorded on a Bruker AV 600, Bruker Advance III 400 MH or a Bruker DPX400 400 MHz or an Agilent 400 spectrometer. Accurate mass measurements (HRMS) were carried out using a Bruker microTOF‐Q™ ESI‐TOF mass spectrometer. Mass spectrometry was performed with a Q‐Tof Premier Waters MALDI quadrupole time‐of‐flight (Q‐TOF) mass spectrometer equipped with Z‐spray electrospray ionization (ESI) and matrix‐assisted laser desorption ionization (MALDI) sources in positive mode with *trans*‐2‐[3‐(4‐*tert*‐butylphenyl)‐2‐methyl‐2‐propenylidene]malononitrile as the matrix. Melting points were measured using an automated melting point meter, SMP50 (Stuart). IR spectra were recorded on a PerkinElmer Spectrum 100 FT‐IR spectrometer. Compounds **7** and **8** and DPM **9**, **10**, and **11** were synthesized and characterized in accordance with literature.^15^, ^16^

**General Procedure A – Porphyrin Condensation** Dipyrromethane (2 equiv.) and aldehyde (2 equiv.) were dissolved in dry DCM (120 mL). The reaction was shielded from light and TFA (cat.) was added. The reaction was stirred at r.t. for 3.5 h. TEA (3 µL) and *p*‐chloranil (6 equiv.) were added and the mixture was heated to 70 °C for 1 h. The product was purified on a silica plug (1:1, DCM/hexane) followed by column chromatography (hexane with 2 % ethyl acetate). The porphyrin was then precipitated from DCM and methanol or recrystallized from DCM and hexane.

**General Procedure B – Endoperoxide Formation:** Parent anthracene strapped porphyrin (1 equiv.) was dissolved in [D]chloroform (3 mL) and irradiated with white light for 15 min. Endoperoxide formation was monitored using ^1^H NMR.

**General Procedure C – Zinc(II) Insertion:** To a solution of parent porphyrin (1 equiv.) in DCM (10 mL) was added Zn(II)acetate (10 equiv.) in methanol and the reaction was stirred at 80 °C for 12 h. The product was purified on a silica plug (DCM) followed by column chromatography (3:1, DCM/hexane) and was recrystallized from DCM/methanol.

**Strapped Porphyrin 19**:** 5,10‐[9',10'‐bis(phenoxymethyl)anthracene]porphyrin**. Porphyrin **19** was synthesized in accordance with general procedure A using dipyrromethane (393 mg, 2.69 mmol), 2,2'‐((anthracene‐9,10‐diylbis(methylene))bis(oxy))dibenzaldehyde (600 mg, 1.35 mmol), DCM (120 mL), and *p*‐chloranil (1.98 g, 8.08 mmol) to yield a purple solid (178 mg, 2.57 × 10^–4^  mol, 19 %). M.p. >200 °C; *R*_f_ = 0.87 (DCM); ^1^H NMR (600 MHz, CDCl_3_): *δ* = 9.64 (s, 2H, C*H*_meso_), 9.04 (d, *J* = 4.3 Hz, 4H, C*H*_beta_), 9.01 (d, *J* = 7.1 Hz, 2H, C*H*_Ar_), 8.84 (d, *J* = 4.3 Hz, 4H, C*H*_beta_), 7.74–7.70 (m, 2H, C*H*_Ar_), 7.67–7.64 (m, 2H, C*H*_Ar_), 7.00 (d, *J* = 7.8 Hz, 2H, C*H*_Ar_), 6.32 (dd, *J* = 6.9, 2.7 Hz, 4H, C*H*_Ar_), 6.25 (dd, *J* = 6.6, 3.1 Hz, 4H, C*H*_Ar_), 4.59 (s, 4H, C*H*_2_), –3.50 ppm (s, 2H, N*H*); ^13^C NMR (600 MHz, CDCl_3_): *δ* = 158.6, 131.4, 130.1, 128.7, 127.1, 126.3, 122.9, 121.6, 120.1, 111.9, 111.8, 103.8, 61.6, 53.4 ppm; UV/Vis (DCM): *λ*_max_ (log *ε*) = 359 (4.41), 378 (4.62), 418 (5.34), 511 (4.06), 543 (3.19), 584 (3.53), 632 nm (2.64); IR (ATR): ν̃ = 3296, 2545, 2162, 1690, 1596, 1575, 1530, 1474, 1444, 1405, 1283, 1217, 1139, 1105, 1058, 1045, 997, 974, 955, 857, 847, 821, 788, 749, 722, 692, 658, 639, 600, 568 cm^–1^; HRMS (MALDI‐TOF) *m/z* calcd. for C_48_H_32_N_4_O_2_ [M]^+^: 696.2525, 696.2524 found.

**Strapped Porphyrin 22**: **5,10‐[9',10'‐dihydro‐9',10'‐epidioxyanthracene]porphyrin**. Porphyrin **22** was synthesized in accordance with general procedure B using porphyrin **19** (19 mg, 2.75 × 10^–5^  mol) to yield a purple solid (20 mg, 2.75 × 10^–5^  mol, quant.). M.p. >200 °C; *R*_f_ = 0.54 (3:1, DCM/hexane); ^1^H NMR (400 MHz, CDCl_3_) *δ* = 9.62 (s, 2H, C*H_meso_*), 9.12 (d, *J* = 4.5 Hz, 4H, C*H_beta_*), 9.06 (m, 2H, C*H_Ar_*), 8.90 (d, *J* = 4.5 Hz, 4H, C*H_beta_*), 7.69 (m, 4H, C*H_Ar_*), 6.78–6.70 (m, 2H, C*H_Ar_*), 5.92 (m, 4H, C*H_Ar_*), 4.88 (m, 4H, C*H_Ar_*), 3.57 (s, 4H, C*H*_2_), –3.24 ppm (br, 1H, N*H*); ^13^C NMR (600 MHz, CDCl_3_) *δ* = 155.9, 132.7, 132.3, 130.8, 130.3, 130.0, 129.0, 124.1, 120.5, 117.9, 110.6, 110.2, 104.5, 78.4, 61.9 ppm; UV/Vis (DCM): *λ*_max_ (log *ε*) = 420 (6.22), 515 (4.88), 545 (3.28), 587 (4.44) 634 nm (2.44); IR (ATR): ν̃ = 1574, 1447, 1408, 1237, 1111, 1042, 976, 957, 908, 882, 787, 746, 892, 829, 848, 571 cm^–1^; HRMS (MALDI‐TOF) *m/z* calcd. for C_48_H_32_N_4_O_4_ [M]^+^: 728.2424, 728.2458 found.

**Strapped Porphyrin 16:** **5,10‐[{9',10'‐bis(phenoxymethyl)anthracene}porphyrinato]zinc(II)**. Porphyrin **16** was synthesized in accordance with general procedure C using porphyrin **19** (50 mg, 7.18 × 10^–5^  mol), DCM (10 mL), Zn(II)acetate (131 mg, 7.18 × 10^–4^  mol), and methanol (2 mL) to yield a pink solid (47 mg, 6.25 × 10^–5^  mol, 87 %). M.p. >200 °C; *R*_f* =*_ 0.88 (3:1, DCM/hexane); ^1^H NMR (400 MHz, CDCl_3_) *δ* = 9.77 (s, 2H, C*H_Ar_*), 9.17 (d, *J* = 4.3 Hz, 4H, C*H_beta_*), 9.05 (d, *J* = 8.6 Hz, 2H, C*H_Ar_*), 9.01 (d, *J* = 4.3 Hz, 4H, C*H_beta_*), 7.73 (t, *J* = 6.2 Hz, 4H, C*H_Ar_*), 7.03–6.95 (m, 2H, C*H_Ar_*), 6.32 (d, *J* = 6.8 Hz, 4H, C*H_Ar_*), 6.13 (dd, *J* = 6.6, 3.2 Hz, 4H, C*H_Ar_*), 4.48 ppm (s, 4H, C*H*_2_); ^13^C NMR (600 MHz, CDCl_3_) *δ* = 158.7, 150.7, 148.2, 132.8, 131.9, 131.5, 129.8, 128.0, 126.6, 126.3, 123.0, 121.3, 120.3, 113.5, 112.2, 105.2, 62.0 ppm; UV/Vis (DCM): *λ*_max_ (log *ε*) = 361 (3.12), 385 (3.23), 433 (4.28), 560 nm (3.01); IR (ATR): ν̃ = 2922, 1884, 1597, 1558, 1445, 1339, 1231, 1109, 1060, 996, 858, 789, 748, 715, 699, 654 cm^–1^; HRMS (MALDI‐TOF) *m/z* calcd. for C_48_H_30_N_4_O_2_Zn [M]^+^: 758.1660, 758.1650 found.

**Strapped Porphyrin 20**:** 5,10‐[{9',10'‐bis(phenoxymethyl)anthracene}‐5,15‐diphenyl]porphyrin**. Porphyrin **20** was prepared in accordance with general procedure A using 2,2'‐(phenylmethylene)bis(1H‐pyrrole) (1.07 g, 4.82 mmol), 2,2'‐((anthracene‐9,10‐diylbis(methylene))bis(oxy))dibenzaldehyde (1.07 g, 2.41 mmol), DCM (350 mL), and *p*‐chloranil (7.06 g, 28.8 mmol) to yield a purple solid (278 mg, 3.31 × 10^–4^  mol, 14 %). M.p. >200 °C; *R*_f_ = 0.84 (3:1, DCM/hexane); ^1^H NMR (400 MHz, CDCl_3_) *δ* = 8.95 (dd, *J* = 7.3, 1.6 Hz, 2H, C*H_Ar_*), 8.77 (d, *J* = 4.7 Hz, 4H, C*H_beta_*), 8.59 (d, *J* = 4.7 Hz, 4H, C*H_beta_*), 7.86 (m, 10H, C*H_Ar_*), 7.75 (t, *J* = 7.8, 3.9 Hz, 2H, C*H_Ar_*), 7.67 (t, *J* = 7.3 Hz, 2H, C*H_Ar_*), 7.06 (d, *J* = 7.7 Hz, 2H, C*H_Ar_*), 6.37 (dd, *J* = 6.8, 3.2 Hz, 4H, C*H_Ar_*), 6.32–6.25 (m, 4H, C*H_Ar_*), 4.71 (s, 4H, C*H*_2_), –2.81 ppm (s, 2H, N*H*); ^13^C NMR (600 MHz, CDCl_3_) *δ* = 169.6, 140.8, 135.5, 131.0, 130.6, 130.0, 128.9, 127.3, 126.7, 122.7, 122.1, 120.3, 112.4, 62.2 ppm; UV/Vis (DCM): *λ*_max_ (log *ε*) = 360 (3.79), 384 (3.92), 431 (4.81), 526 (3.50), 564 (3.19), 600 (3.10) 658 nm (2.87); IR (ATR): ν̃ = 3359, 1878, 1588, 1445, 1407, 1307, 1107, 983, 966, 908, 882, 858, 794, 748, 711, 600, 575 cm^–1^; HRMS (MALDI‐TOF) *m/z* calcd. for C_60_H_40_N_4_O_2_ [M]^+^: 848.3141, 848.3151 found.

**Strapped Porphyrin 23**:** 5,10‐[{9',10'‐bis(phenoxymethyl)‐9',10'‐dihydro‐9',10'‐epidioxyanthracene}‐5,15‐diphenyl]porphyrin**. Porphyrin **23** was synthesized in accordance with general procedure B using porphyrin **20** (20 mg, 2.27 × 10^–5^  mol) to yield a purple solid (20 mg, 2.27 × 10^–5^  mol, quant.). M.p. >200 °C; *R*_f_ = 0.74 (3:1, DCM/hexane);^ 1^H NMR (400 MHz, CDCl_3_) *δ* = 8.98 (d, *J* = 7.0 Hz, 2H, C*H_Ar_*), 8.83 (d, *J* = 4.8 Hz, 4H, C*H_beta_*), 8.70 (d, *J* = 4.7 Hz, 4H, C*H_beta_*), 7.92 (s, 10H, C*H_Ar_*), 7.70 (t, *J* = 7.7 Hz, 4H, C*H_Ar_*), 6.81 (d, *J* = 7.8 Hz, 2H. C*H_Ar_*), 6.04 (d, *J* = 3.3 Hz, 4H, C*H_Ar_*), 5.09 (s, 4H, C*H_Ar_*), 3.72 (s, 4H, C*H*_2_), –2.57 ppm (s, 12H, N*H*); ^13^C NMR (600 MHz, CDCl_3_) *δ* = 155.9, 140.1, 133.6, 131.3, 130.4, 130.2, 130.0, 129.1, 123.6, 122.7, 122.3, 122.2, 120.6, 118.6, 117.2, 112.4, 110.3, 78.6, 62.0 ppm; UV/Vis (DCM): *λ*_max_ (log *ε*) = 433 (5.03), 528 (3.77), 567 (3.67), 601 (3.65), 657 nm (3.48); IR (ATR): ν̃ = 3273, 3066, 2932, 3883, 1787, 1688, 1598, 1560, 1466, 1447, 1241, 1110, 983, 967, 905, 792, 750, 730 cm^–1^; HRMS (ESI) *m/z* calcd. for C_60_H_41_N_4_O_4_ [M + H]^+^: 881.3050, 881.3125 found.

**Strapped Porphyrin 17**:** 5,10‐[{9',10'‐bis(phenoxymethyl)anthracene}‐5,15‐diphenylporphyrinato]zinc(II)**. Porphyrin **17** was synthesized in accordance with general procedure C using porphyrin **20** (100 mg, 1.18 × 10^–4^  mol), DCM (6 mL), Zn(II)acetate (424 mg, 2.34 × 10^–3^  mol), and methanol (1 mL) to yield a purple/pink solid (50 mg, 5.49 × 10^–5^  mol_,_ 47 %). M.p. >200 °C; *R*_f_ = 0.88 (3:1, DCM/hexane); ^1^H NMR (400 MHz, CDCl_3_) *δ* = 8.91 (dd, *J* = 7.0, 1.8 Hz, 2H, C*H_Ar_*), 8.83 (d, *J* = 4.6 Hz, 4H, C*H_beta_*), 8.65 (d, *J* = 4.6 Hz, 4H, C*H_beta_*), 7.67 (ddd, *J* = 21.8, 10.2, 6.1 Hz, 14H, C*H_Ar_*), 6.96 (d, *J* = 7.4 Hz, 2H, C*H_Ar_*), 6.34–6.29 (m, 4H, C*H_Ar_*), 6.15 (dd, *J* = 6.7, 2.9 Hz, 4H, C*H_Ar_*), 4.45 ppm (s, 4H, C*H*_2_); ^13^C NMR (600 MHz, CDCl_3_) *δ* = 158.7, 150.2, 148.4, 142.7, 133.1, 131.9, 130.9, 129.8, 128.0, 127.1, 126.8, 126.2, 122.8, 121.6, 120.5, 119.8, 114.5, 112.8, 62.6 ppm; UV/Vis (DCM): *λ*_max_ (log *ε*) = 357 (4.08), 380 (4.28), 420 (5.16), 548 nm (3.87); IR (ATR): ν̃ = 1638, 1878, 15889 1449, 1230, 1107, 1053, 993, 848, 745, 891, 845, 579 cm^–1^; HRMS (MALDI‐TOF) *m/z* calcd. for C_60_H_38_N_4_O_2_Zn [M]^+^: 910.2286, 910.2273 found.

**Strapped Porphyrin 21**: **5,10‐[{9',10'‐bis(phenoxymethyl)anthracene}‐5,15‐bis(4‐bromophenyl)]porphyrin**. Porphyrin **21** was synthesized in accordance with general procedure A using 2,2'‐((4‐bromophenyl)methylene)bis(1H‐pyrrole) (1.29 g, 4.31 mmol), 2,2'‐((anthracene‐9,10‐diylbis(methylene))bis(oxy))dibenzaldehyde (959 mg, 2.15 mmol), DCM (300 mL), and *p*‐chloranil (7.06 g, 28.8 mmol) to yield a purple solid (328 mg, 3.31 × 10^–4^  mol, 15 %). M.p. >200 °C; *R*_f_ = 0.84 (3:1, DCM/hexane); ^1^H NMR (400 MHz, CDCl_3_) *δ* = 8.95 (dd, *J* = 7.3, 1.7 Hz, 2H, C*H_Ar_*), 8.77 (d, *J* = 4.7 Hz, 4H, C*H_beta_*), 8.62 (d, *J* = 4.7 Hz, 4H, C*H_beta_*), 7.77–7.70 (m, 8H, C*H_Ar_*), 7.66 (t, *J* = 7.4 Hz, 2H, C*H_Ar_*), 7.06 (d, *J* = 7.5 Hz, 2H, C*H_Ar_*), 6.45–6.35 (m, 8H, C*H_Ar_*), 4.74 (s, 4H, C*H*_2_), –2.85 ppm (s, 2H, N*H*); ^13^C NMR (600 MHz, CDCl_3_) *δ* = 169.6, 158.9, 140.8, 134.2, 130.8, 128.9, 127.3, 126.8, 122.8, 122.1, 120.2, 112.3, 77.3, 77.1, 76.7, 62.2 ppm; UV/Vis (DCM): *λ*_max_ (log *ε*) = 361 (3.08), 382 (3.16), 432 (4.01), 527 (2.71), 565 (2.41), 601 (2.30), 658 nm (2.07); IR (ATR): ν̃ = 3358, 1888, 1877, 1588, 1258, 1234, 1106, 984, 908, 792, 754, 710 cm^–1^; HRMS (MALDI‐TOF) *m/z* calcd. for C_60_H_40_N_4_O_2_ [M]^+^: 1004.1361, 1004.1334 found.

**Strapped Porphyrin 24: 5,10‐[{9',10'‐bis(phenoxymethyl)‐9',10'‐dihydro‐9',10'‐epidioxyanthracene}‐5,15‐bis(4‐bromophenyl)}]porphyrin**. Porphyrin 24 was synthesized in accordance with general procedure B using porphyrin 21 (20 mg, 1.99 × 10^–5^  mol) to yield a purple solid (21 mg, 1.99 × 10^–5^  mol, quant.). M.p. >200 °C; *R*_f_ = 0.66 (3:1, DCM/hexane); ^1^H NMR (400 MHz, CDCl_3_) *δ* = 8.98 (dd, *J* = 6.9, 2.0 Hz, 2H, C*H_Ar_*), 8.81 (d, *J* = 4.8 Hz, 4H, C*H_beta_*), 8.73 (d, *J* = 4.7 Hz, 4H, C*H_beta_*), 7.83–7.72 (m, 8H, C*H_Ar_*), 7.71–7.65 (m, 4H, C*H_Ar_*), 6.84–6.78 (m, 2H, C*H_Ar_*), 6.08 (dd, *J* = 5.7, 3.2 Hz, 4H, C*H_Ar_*), 5.10 (dd, *J* = 5.5, 3.3 Hz, 4H, C*H_Ar_*), 3.73 (s, 4H. C*H*_2_), –2.53 ppm (s, 2H, N*H*); ^13^C NMR (600 MHz, CDCl_3_) *δ* = 156.0, 144.7, 141.2, 133.5, 130.9, 130.2, 129.2, 127.5, 127.2, 123.7, 120.5, 120.6, 118.6, 112.0, 110.3, 78.7, 62.0 ppm; UV/Vis (DCM): *λ*_max_ (log *ε*) = 434 (5.46), 529 (4.10), 566 (3.81), 604 (3.81), 660 nm (3.50); IR (ATR): ν̃ = 3359, 3081, 2544, 1790, 1689, 1679, 1570, 1464, 1408, 1011, 983, 966, 884, 792, 751, 712 cm^–1^; HRMS (APCI) *m/z* calcd. for C_60_H_39_Br_2_N_4_O_4_ [M + H]^+^: 1037.1334, 1037.1334 found.

**Strapped Porphyrin 18**: **5,10‐[{9',10'‐bis(phenoxymethyl)anthracene}‐5,15‐bis(4‐bromophenyl)porphyrinato]zinc(II)**. Porphyrin **18** was synthesized in accordance with general procedure C using porphyrin **21** (50 mg, 4. 98 × 10^–5^  mol) dissolved in DCM (6 mL), Zn(II)acetate (140 mg, 4.98 × 10^–4^  mol), and methanol (1 mL) to yield a purple solid (52 mg, 4.90 × 10^–5^  mol, 98 %); M.p. >200 °C; *R*_f_ = 0.87 (3:1, DCM/hexane); ^1^H NMR (400 MHz, CDCl_3_) *δ* = 8.92 (dd, *J* = 7.1, 2.0 Hz, 2H, C*H_Ar_*), 8.87 (d, *J* = 4.6 Hz, 4H, C*H_beta_*), 8.63 (d, *J* = 4.6 Hz, 4H, C*H_beta_*), 7.84 (s, 6H, C*H_Ar_*), 7.68 (tdd, *J* = 14.9, 10.6, 4.2 Hz, 6H, C*H_Ar_*), 6.99 (d, *J* = 7.9 Hz, 2H, C*H_Ar_*), 6.34 (dd, *J* = 6.9, 3.1 Hz, 4H, C*H_Ar_*), 6.20 (dd, *J* = 6.8, 3.2 Hz, 4H, C*H_Ar_*), 4.50 ppm (s, 4H, C*H*_2_); ^13^C NMR (600 MHz, CDCl_3_) *δ* = 158.7, 150.5, 148.2, 141.6, 140.7, 135.4, 133.0, 131.7, 131.3, 130.0, 129.6, 128.2, 128.0, 126.8, 126.6, 126.2, 125.3, 122.7, 121.9, 121.8, 120.6, 118.3, 115.1, 113.1, 62.7, 29.7 ppm; UV/Vis (DCM): *λ*_max_ (log *ε*) = 362 (3.96), 384 (4.05), 432 (5.11), 560 nm (3.84); IR (ATR): ν̃ = 2922, 1680, 1558, 1482, 1445, 1230, 1109, 1068, 999, 790, 717, 749, 645 cm^–1^; HRMS (MALDI‐TOF) *m/z* calcd. for C_60_H_36_N_4_O_2_Br_2_Zn [M]^+^: 1066.0496, 1066.0493 found.

**Supporting Information** (see footnote on the first page of this article): Spectroscopic data of all compounds and X‐ray crystallographic data (NSD, crystal structures, packing, bond lengths and angles).

CCDC 1981333 (for **19**), and 1981332 (for **22**) contain the supplementary crystallographic data for this paper. These data can be obtained free of charge from The Cambridge Crystallographic Data Centre.

## Supporting information

Supporting Information

## References

[1] C. Moureu , C. Dufraisse and P. M. Dean , C. R. Acad. Sci, 1926, 182, 1584–1587.

[2] a) C. Flors , M. J. Fryer , J. Waring , B. Reeder , U. Bechtold , P. M. Mullineaux , S. Nonell , M. T. Wilson and N. R. Baker , J. Exp. Bot, 2006, 57, 1725–1734; 16595576 10.1093/jxb/erj181

[3] a) S. Benz , S. Nötzli , J. S. Siegel , D. Eberli and H. J. Jessen , J. Med. Chem, 2013, 56, 10171–10182; 24299550 10.1021/jm4016137

[4] a) I. Saito , R. Nagata and T. Matsuura , J. Am. Chem. Soc, 1985, 107, 6329–6334;

[5] W. Fudickar , A. Fery and T. Linker , J. Am. Chem. Soc, 2005, 127, 9386–9387. 15984863 10.1021/ja0517015

[6] M. O. Senge , Chem. Commun, 2006, 243–256. 10.1039/b511389j 16391725

[7] a) M. Ravikanth and T. K. Chandrashekar , Struct. Bonding (Berlin), 1995, 82, 105–188;

[8] a) M. Newcomb , R. Zhang , R. E. P. Chandrasena , J. A. Halgrimson , J. H. Horner , T. M. Makris and S. G. Sligar , J. Am. Chem. Soc, 2006, 128, 4580–4581; 16594688 10.1021/ja060048y PMC2536593

[9] a) M. Roucan , M. Kielmann , S. J. Connon , S. S. R. Bernhard and M. O. Senge , Chem. Commun, 2017, 54, 26–29; 10.1039/c7cc08099a 29226923

[10] a) T. P. Wijesekera , J. B. Paine III , D. Dolphin , F. W. B. Einstein and T. Jones , J. Am. Chem. Soc, 1983, 105, 6747–6749;

[11] J. Weiser and H. A. Staab , Angew. Chem. Int. Ed. Engl, 1984, 23, 623–625;

[12] a) T. G. Traylor , M. J. Mitchell , S. Tsuchiya , D. H. Campbell , D. V. Stynes and N. Koga , J. Am. Chem. Soc, 1981, 103, 5234–5236;

[13] A. Osuka , F. Kobayashi and K. Maruyama , Bull. Chem. Soc. Jpn, 1991, 64, 1213–1225.

[14] a) A. C. Gehrold , T. Bruhn , H. Schneider , U. Radius and G. Bringmann , Org. Lett, 2015, 17, 210–213; 25556288 10.1021/ol503286s

[15] a) H. A. Muathen , N. A. M. Aloweiny and A. H. M. Elwahy , J. Heterocycl. Chem, 2009, 46, 656–663;

[16] a) Q. M. Wang and D. W. Bruce , Synlett, 1995, 12, 1267–1268;

[17] J. M. Aubry , C. Pierlot , J. Rigaudy and R. Schmidt , Acc. Chem. Res, 2003, 36, 668–675. 12974650 10.1021/ar010086g

[18] D. Reddy , T. K. Chandrashekar and H. van Willigen , Chem. Phys. Lett, 1993, 202, 120–126.

[19] a) A. D. Adler , F. R. Longo and W. Shergalis , J. Am. Chem. Soc, 1964, 86, 3145–3149;

[20] C. Brückner , J. J. Posakony , C. K. Johnson , R. W. Boyle , B. R. James and D. Dolphin , J. Porphyrins Phthalocyanines, 1998, 2, 455–465.

[21] a) K. J. Brunings and A. H. Corwin , J. Am. Chem. Soc, 1942, 64, 593–600;

[22] A. D. Bond , N. Feeder , J. E. Redman , S. J. Teat and J. K. M. Sanders , Cryst. Growth Des, 2002, 2, 27–39.

[23] W. Jentzen , J. G. Ma and J. A. Shelnutt , Biophys. J, 1998, 74, 753–763. 9533688 10.1016/S0006-3495(98)74000-7 PMC1302556

[24] W. Jentzen , M. C. Simpson , J. D. Hobbs , X. Song , T. Ema , N. Y. Nelson , C. J. Medforth , K. M. Smith , M. Veyrat , M. Mazzanti , R. Ramasseul , J. C. Marchon , T. Takeuchi , W. A. Goddard and J. A. Shelnutt , J. Am. Chem. Soc, 1995, 117, 11085–11097. 27275517 10.1021/ja00150a008

[25] W. Jentzen , X. Z. Song and J. A. Shelnutt , J. Phys. Chem. B, 1997, 101, 1684–1699.

[26] a) S. Neidle and M. B. Hursthouse , Acta Crystallogr., Sect. B, 1978, 34, 2509–2514;

[27] a) H. Hope , Prog. Inorg. Chem, 1994, 1–19;

[28] Saint, Version 8.37a; Bruker AXS, Inc.: Madison, WI, 2013.

[29] SADABS, version **2016**/2; Bruker AXS, Inc.: Madison, WI, 2014.

[30] APEX3, Version **2016**9–0; Bruker AXS, Inc.: Madison, WI, 2016.

[31] O. V. Dolomanov , L. J. Bourhis , R. J. Gildea , J. A. K. Howard and H. Puschmann , J. Appl. Crystallogr, 2009, 42, 339–341. 10.1107/S0021889811041161 PMC3236671 22199401

[32] G. Sheldrick , Acta Crystallogr., Sect. A, 2015, 71, 3–8. 10.1107/S2053273314026370 PMC4283466 25537383

[33] J. Schindler , S. Kupfer , A. A. Ryan , K. J. Flanagan , M. O. Senge and B. Dietzek , Coord. Chem. Rev, 2018, 360, 1–16.

[34] NSDGUI, Version 1.3 alpha; Sandia National Laboratory: New Mexico, 2001.

