# Supplementary material for: Short‐Chained Anthracene Strapped Porphyrins and their Endoperoxides
Source: European J Org Chem. 2020 Apr 20;2020(18):2735–44. doi: 10.1002/ejoc.202000283 (PMC7319435; doi:10.1002/ejoc.202000283)
Supplement: Supplementary file 1 — Supporting Information [file EJOC-2020-2735-s001.pdf]

European Journal of Organic Chemistry

Supporting Information

Short-Chained Anthracene Strapped Porphyrins and their Endoperoxides

Susan Callaghan, Keith J. Flanagan, John E. O'Brien, and Mathias O. Senge*

Contents

1. Supporting X-ray Data 2

2. NMR spectra 12

2.1 NMR spectra of porphyrin 19 12

2.2 NMR spectra of porphyrin 22 14

2.3 NMR spectra of porphyrin 16 16

2.4 NMR spectra of 20 18

2.5 NMR spectra of 23 20

2.6 NMR spectra of 17 22

2.7 NMR spectra of 21 24

2.8 NMR spectra of 24 26

2.9 NMR spectra of 18 28

1. Supporting X-ray Data

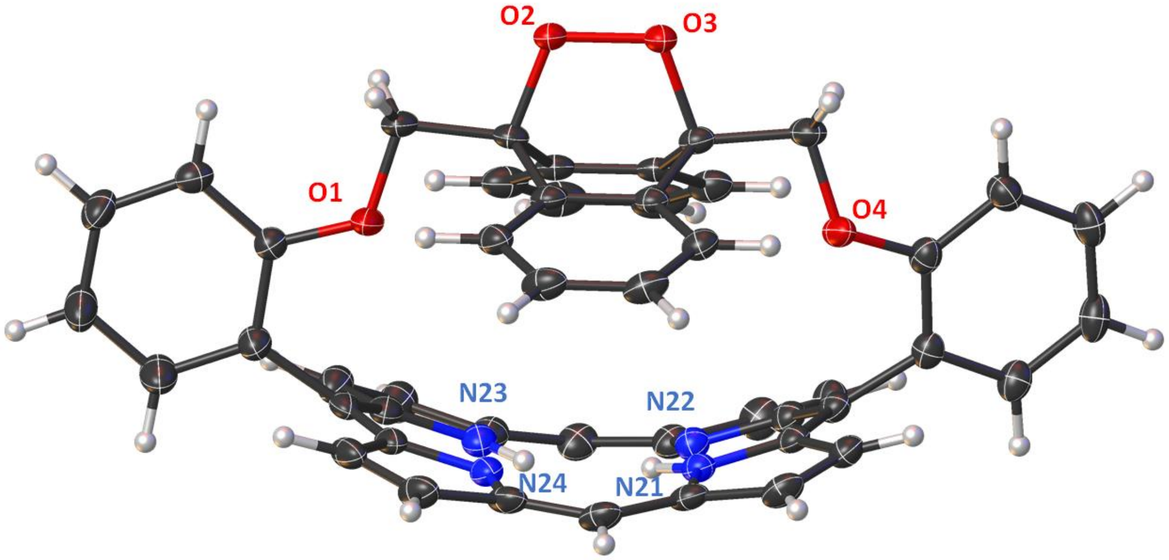

Figure S1: Molecular structure (side view) of **22** showing the curved porphyrin ring and epoxide strap (thermal displacement given as 50%). Solvent molecules and minor disorder have been omitted for clarity.

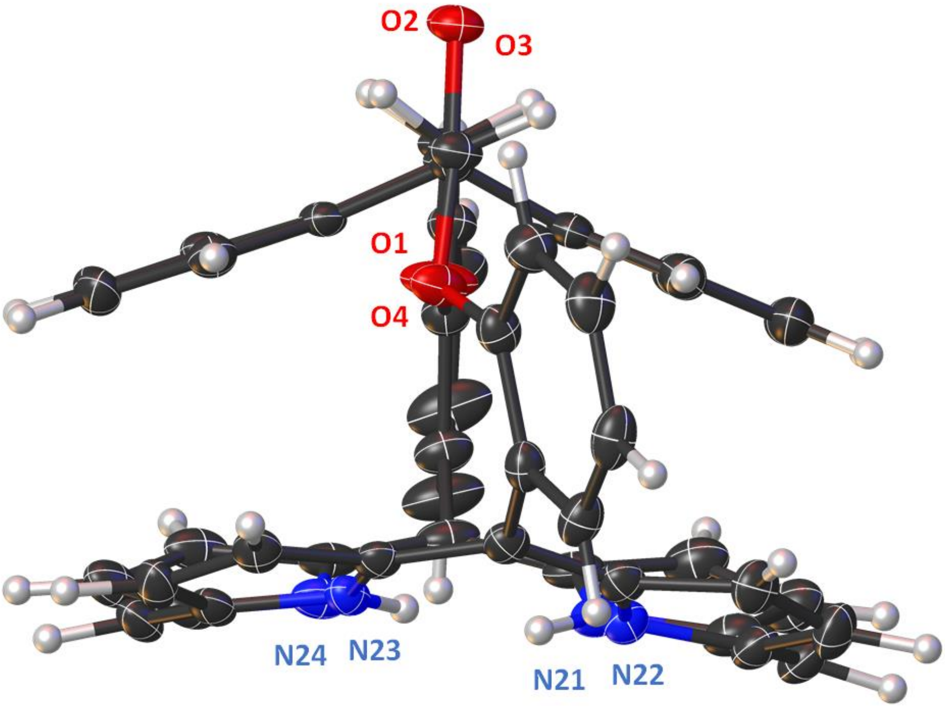

Figure S2: Molecular structure (side view) of **22** showing the bent epoxide strap (thermal displacement given as 50%). Solvent molecules and minor disorder have been omitted for clarity.

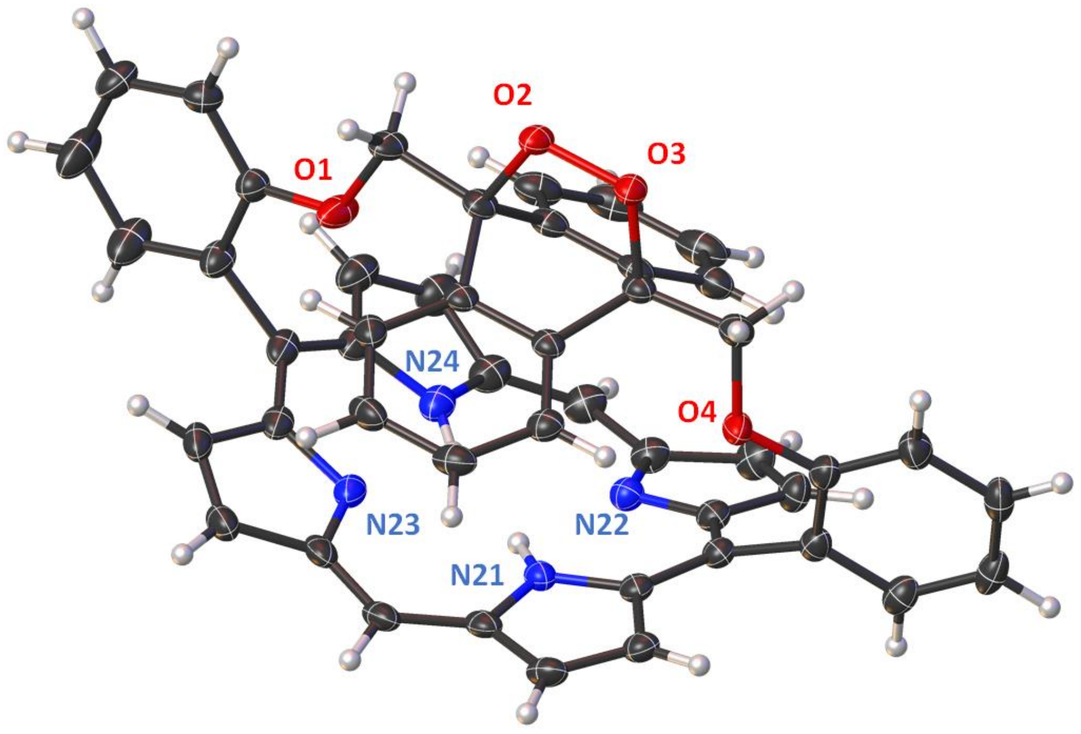

Figure S3: Molecular structure of **22** (thermal displacement given as 50%). Solvent molecules and minor disorder have been omitted for clarity.

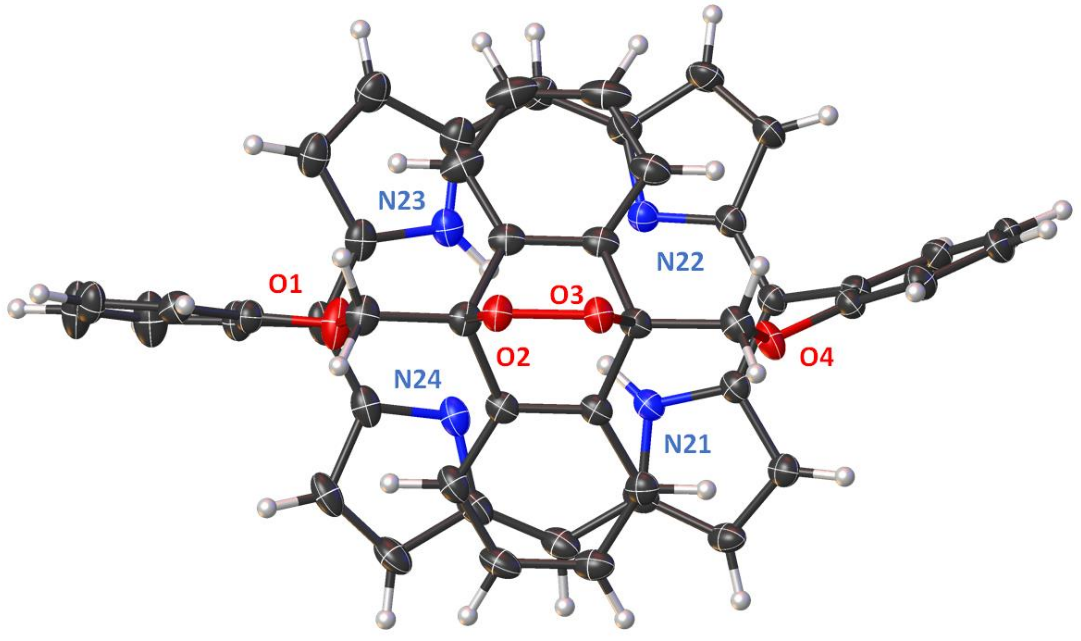

Figure S4: Molecular structure (top view) of **22** showing the bend in the epoxide strap (thermal displacement given as 50%). Solvent molecules and minor disorder have been omitted for clarity.

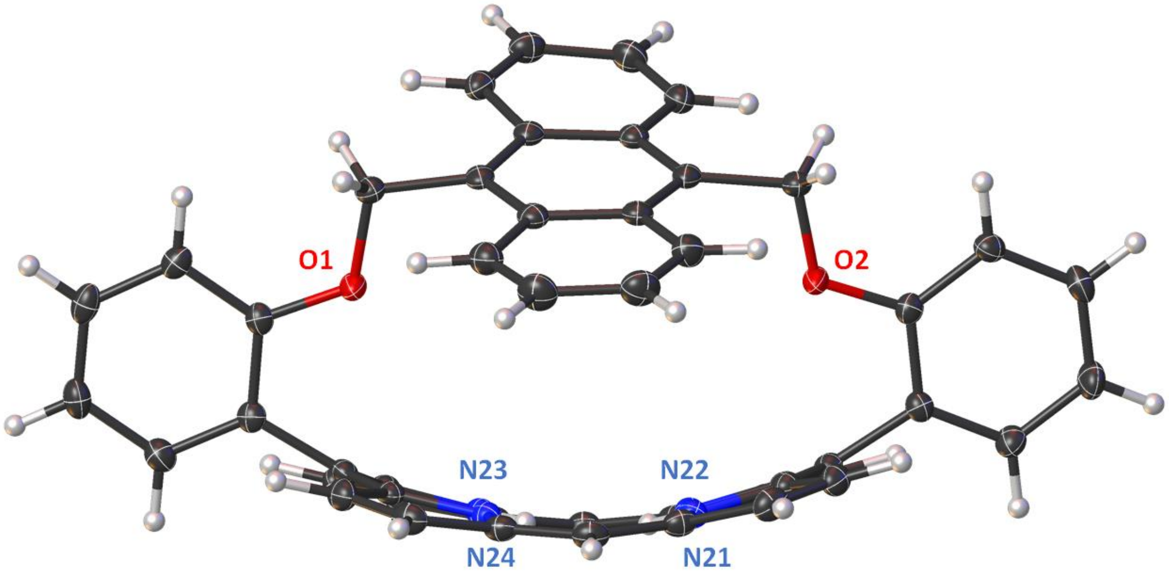

Figure S5: Molecular structure (side view) of **19** showing the curved porphyrin ring and anthracene strap (thermal displacement given as 50%). Solvent molecules and minor disorder have been omitted for clarity.

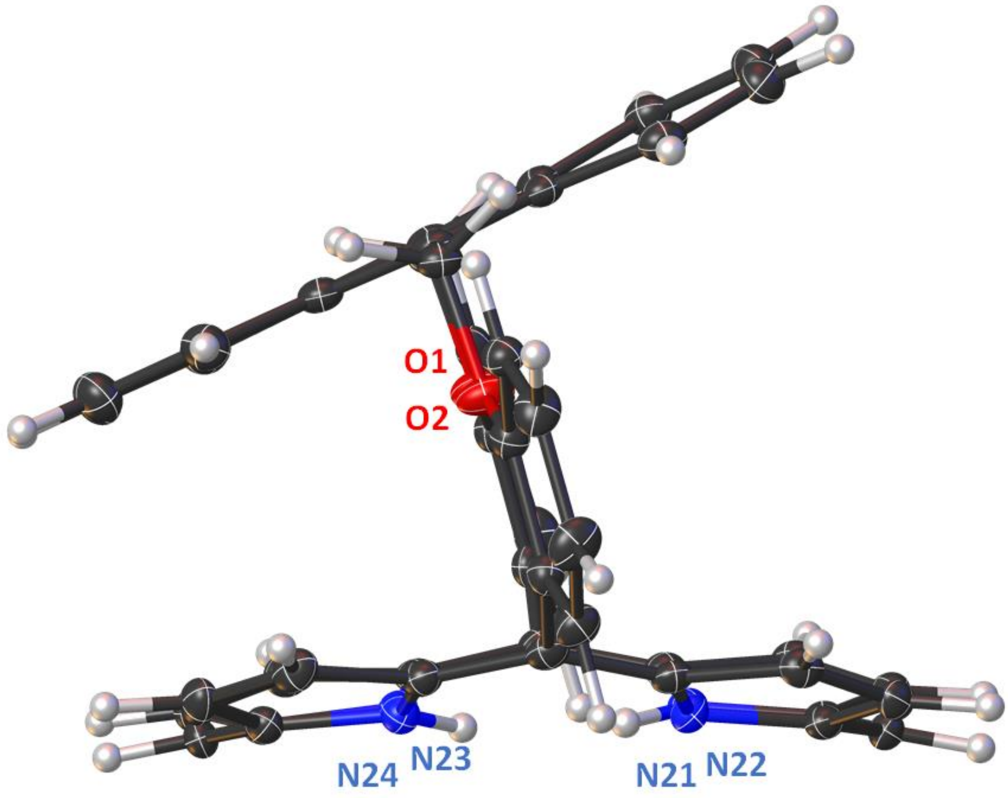

Figure S6: Molecular structure (side view) of **19** showing the angle between the porphyrin macrocycle and the anthracene strap (thermal displacement given as 50%). Solvent molecules and minor disorder have been omitted for clarity.

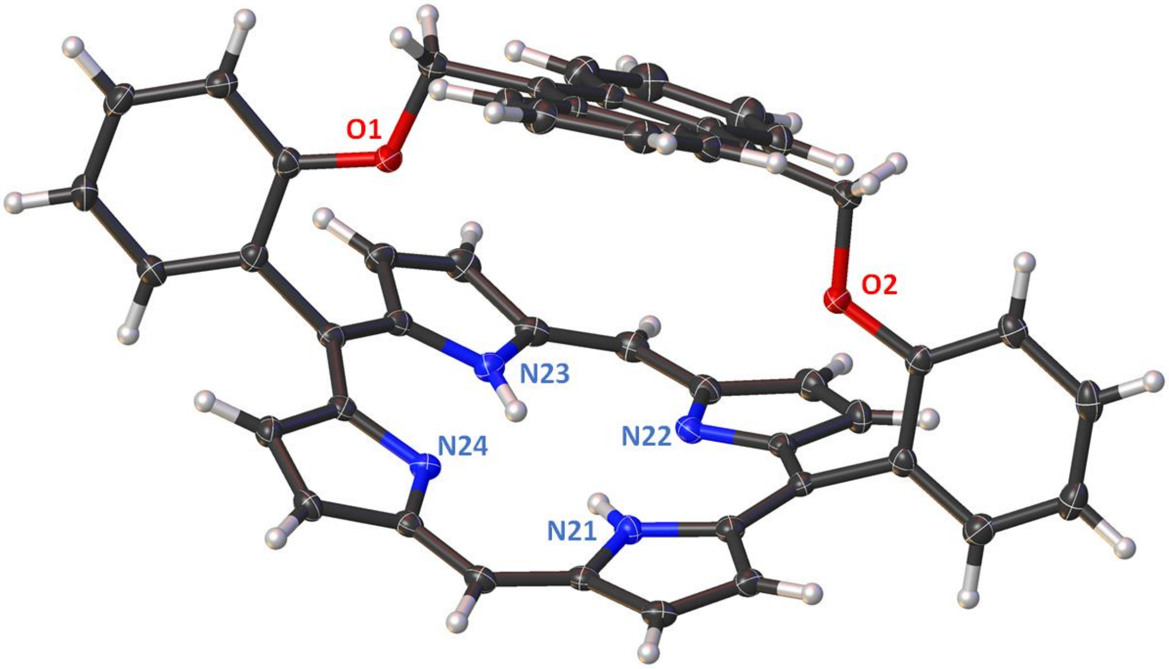

Figure S7: Molecular structure of **19** (thermal displacement given as 50%). Solvent molecules and minor disorder have been omitted for clarity.

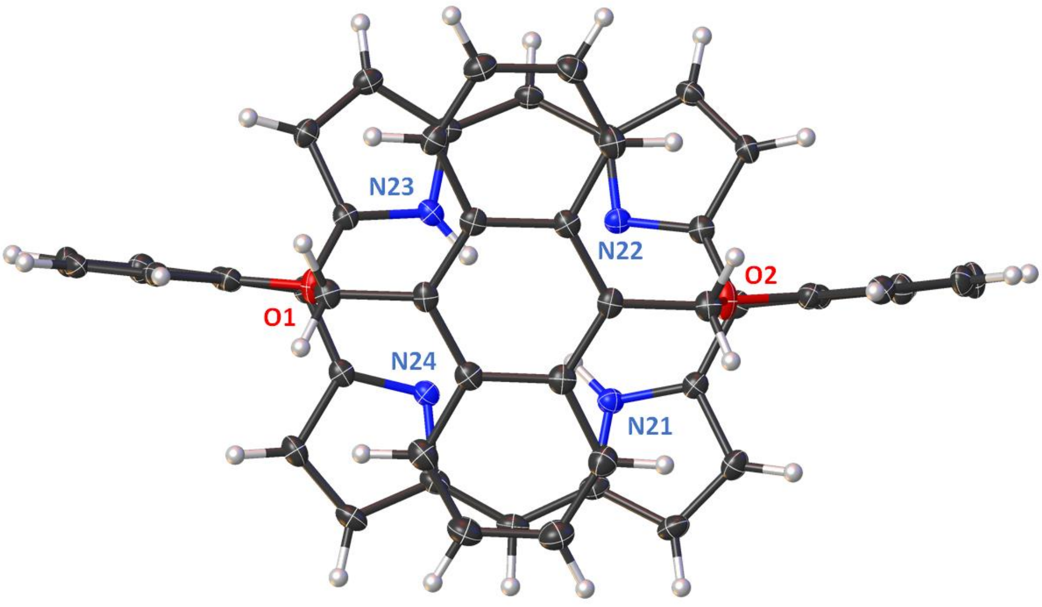

Figure S8: Molecular structure (top view) of **19** showing the curved present in the anthracene strap (thermal displacement given as 50%). Solvent molecules and minor disorder have been omitted for clarity.

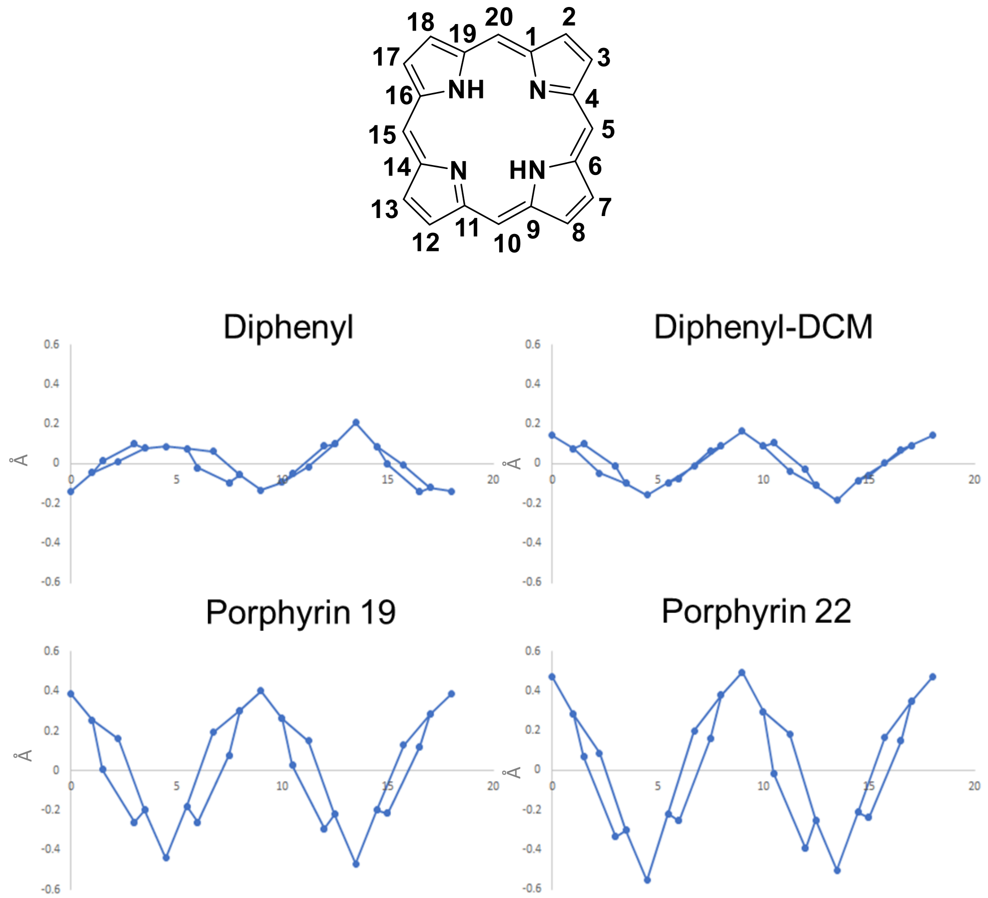

Figure S9: Numbered porphyrin core structure and view of skeletal deviation plots (C20 to C20) from the crystal structure of porphyrins (diphenyl, diphenyl-DCM, porphyrin **19,** and porphyrin **22**) base off the atom displacement from the 24-atom mean plane.

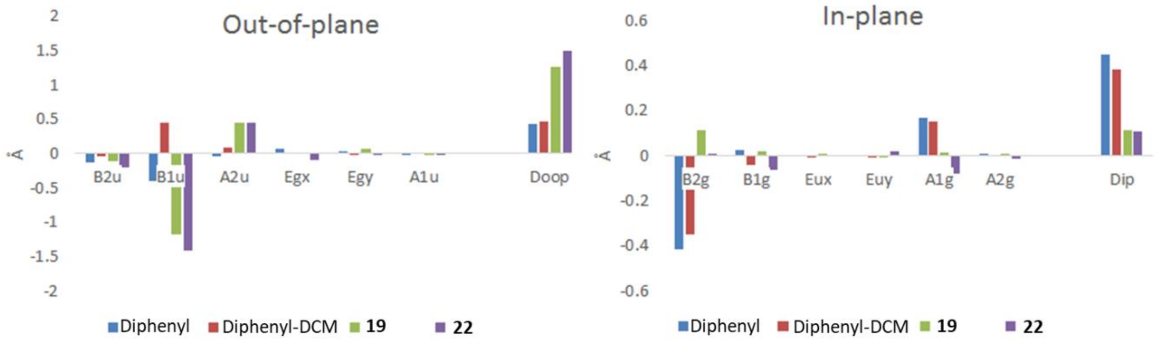

Figure S10: Out-of-plane (left) and in-plane (right) distortion modes from the crystal structure of porphyrins (diphenyl, diphenyl-DCM, porphyrin **19,** and porphyrin **22**) based off the normal-coordinate structural decomposition.

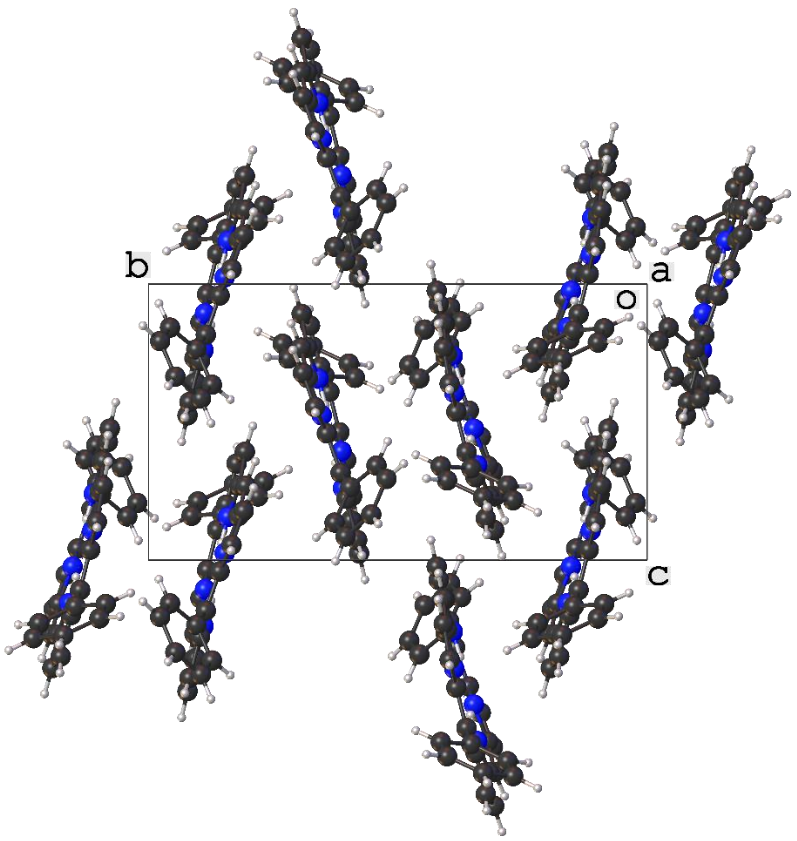

Figure S11: Moiety packing (*a*-axis) of **diphenyl** drawn isotropically.

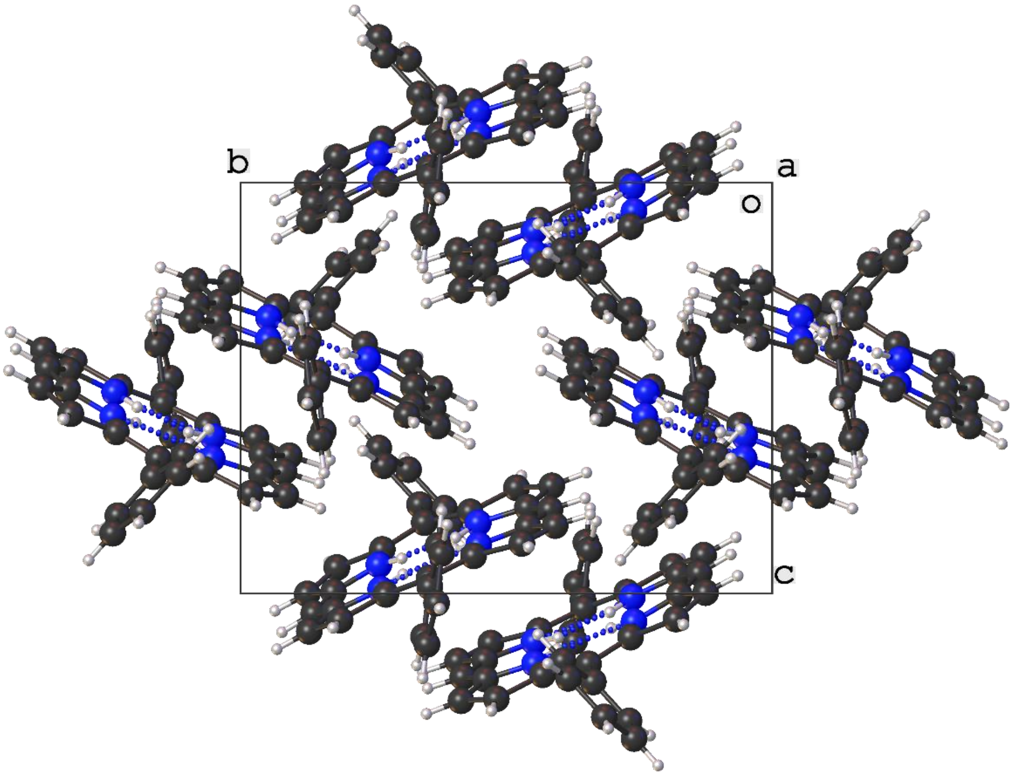

Figure S12: Moiety packing (*a*-axis) of **diphenyl-DCM** drawn isotropically. Solvent molecules have been omitted for clarity.

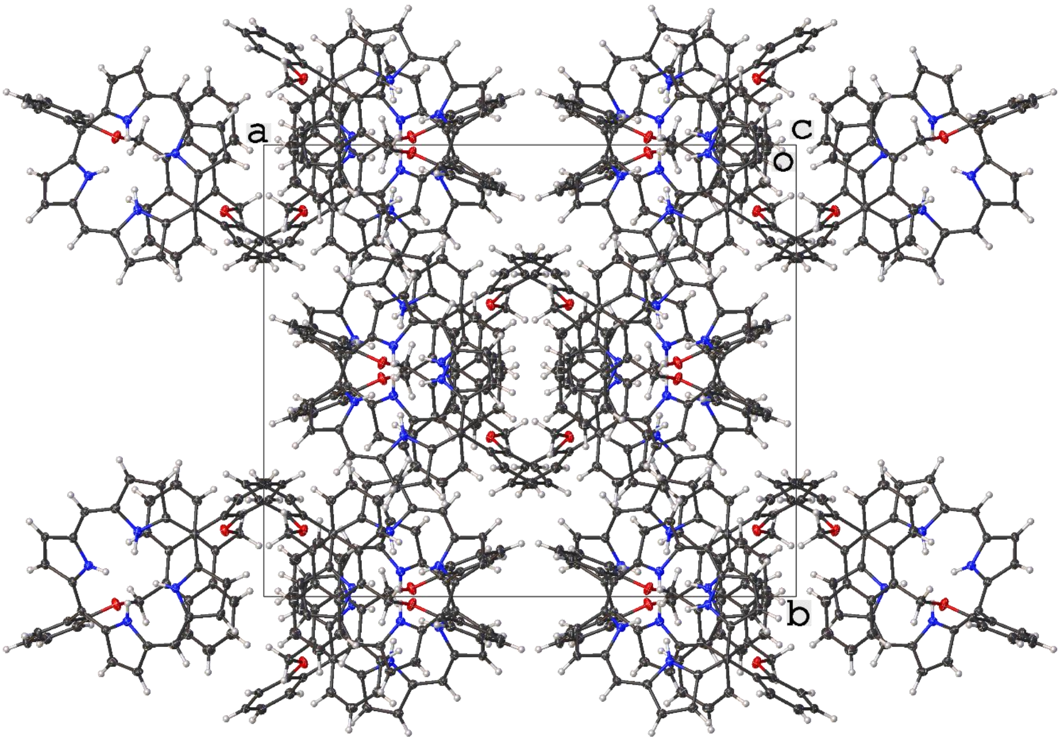

Figure S13: Moiety packing (*c*-axis) of **19** (thermal displacement given as 50%). Solvent molecules and minor disorder have been omitted for clarity.

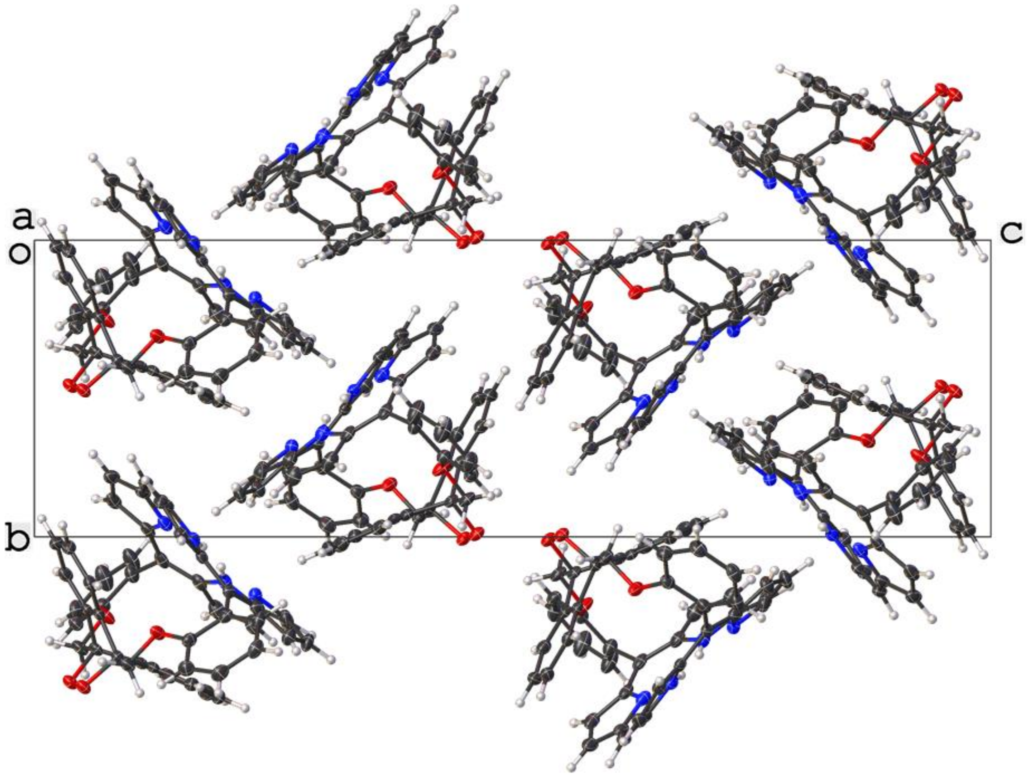

Figure S14: Moiety packing (*a*-axis) of **22** (thermal displacement given as 50%). Solvent molecules and minor disorder have been omitted for clarity.

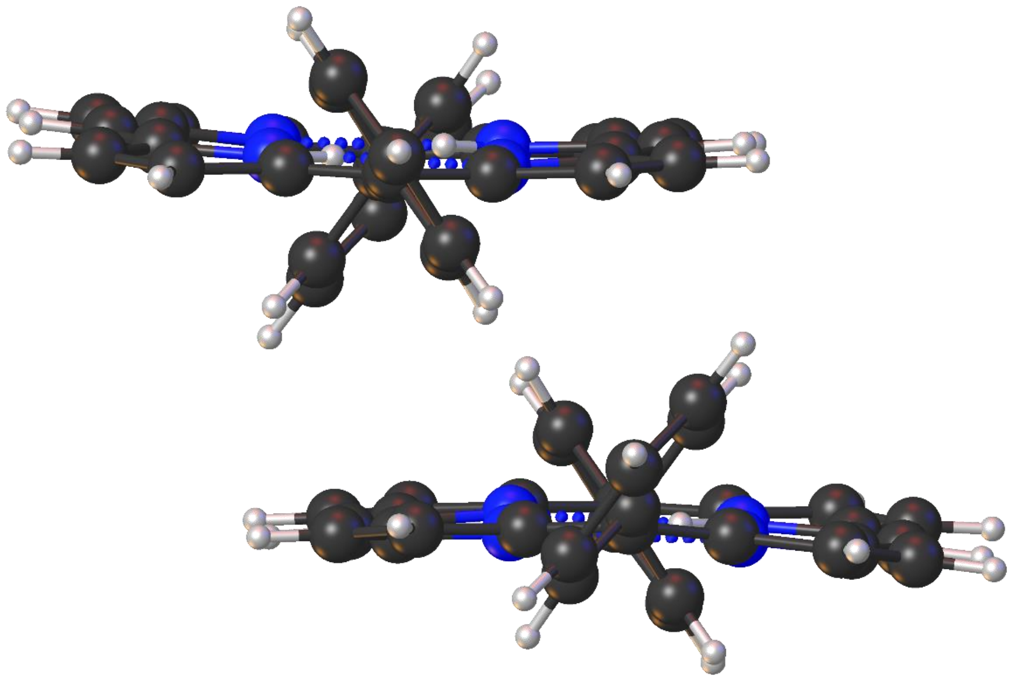

Figure S15: Stacking interactions seen between **diphenyl** (left) and **diphenyl-DCM** (right).

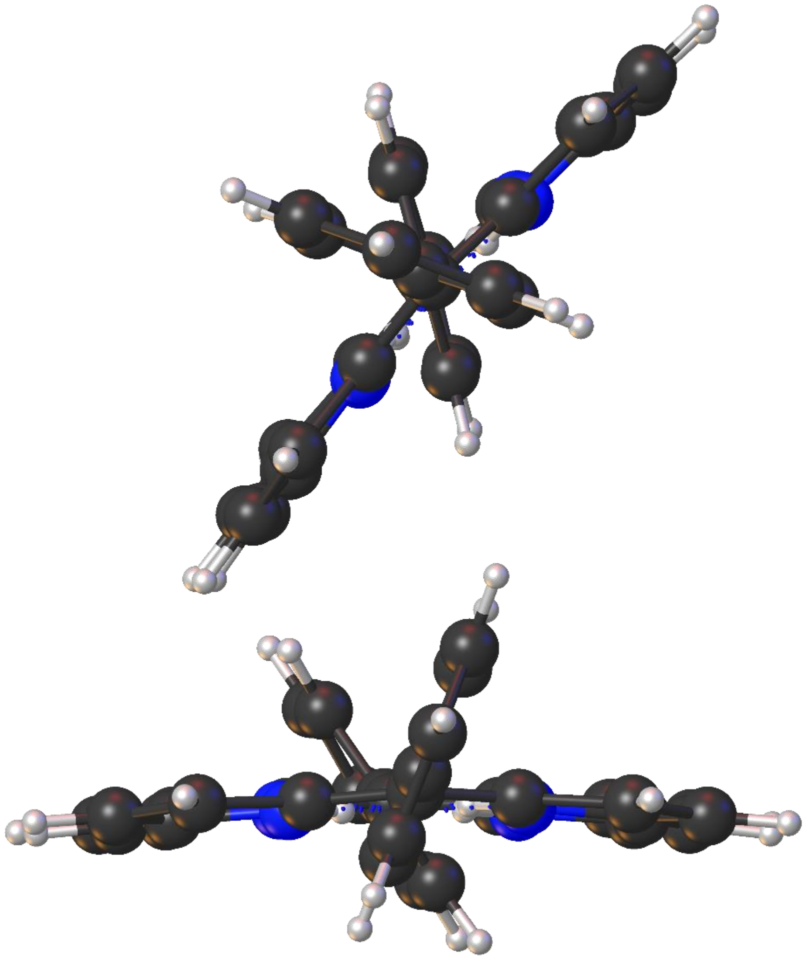

Figure S16: Stacking interactions seen between **diphenyl** (left) and **diphenyl-DCM** (right).

Table S1: Details of XRD data refinement.

Porphyrin	**19**	**22**
Empirical formula	$C_{49}H_{33}Cl_3N_4O_2$	$C_{49}H_{34}Cl_2N_4O_4$
Formula weight	816.14	813.70
Temperature/K	100.01	100.0
Crystal system	monoclinic	monoclinic
Space group	C2/c	$P2_1/c$
a/Å	23.2809(8)	13.2956(14)
b/Å	19.1904(7)	9.4478(10)
c/Å	17.6664(6)	30.565(3)
α/°	90	90
β/°	103.647(2)	93.800(2)
γ/°	90	90
Volume/Å^3	7670.0(5)	3831.0(7)
Z	8	4
D_{calc} *g/cm*^{3}	1.414	1.411
μ/mm^{-1}	0.288	0.224
F(000)	3376.0	1688.0
Crystal size/mm^3	0.2×0.14×0.1	0.5 × 0.2 × 0.1
Radiation	MoK_{α}	MoK_{α}
Wavelength/Å	λ = 0.71073	λ = 0.71073
2θ/°	2.782–51.996	2.670–50.496
Reflections collected	99728	75683
Independent reflections	7541	6941
R_{int}	0.0555	0.0436
R_{sigma}	0.0250	0.0203
Restraints	2	0
Parameters	523	532
GooF	1.028	1.079
R_1 $[I > 2\sigma(I)]$	0.0435	0.0551
wR_2 $[I > 2\sigma(I)]$	0.1082	0.1321
R_1 *[all data]*	0.0612	0.0693

wR_2 [all data]	0.1188	0.1422
Largest peak/e Å^{-3}	0.60	1.19
Deepest hole/e Å^{-3}	-0.61	-0.91

2. NMR spectra

2.1 NMR spectra of porphyrin 19

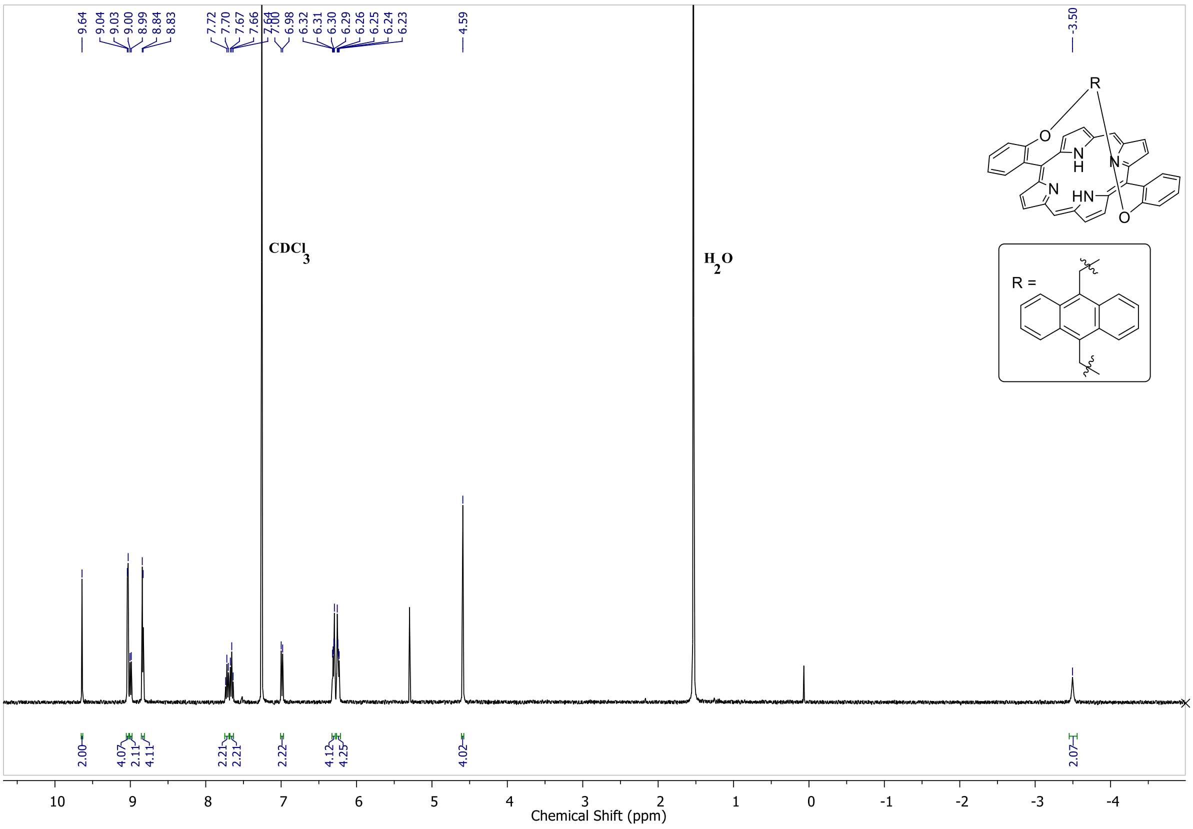

Figure S17: ^{1}H NMR spectrum of porphyrin **19** in $CDCl_3$.

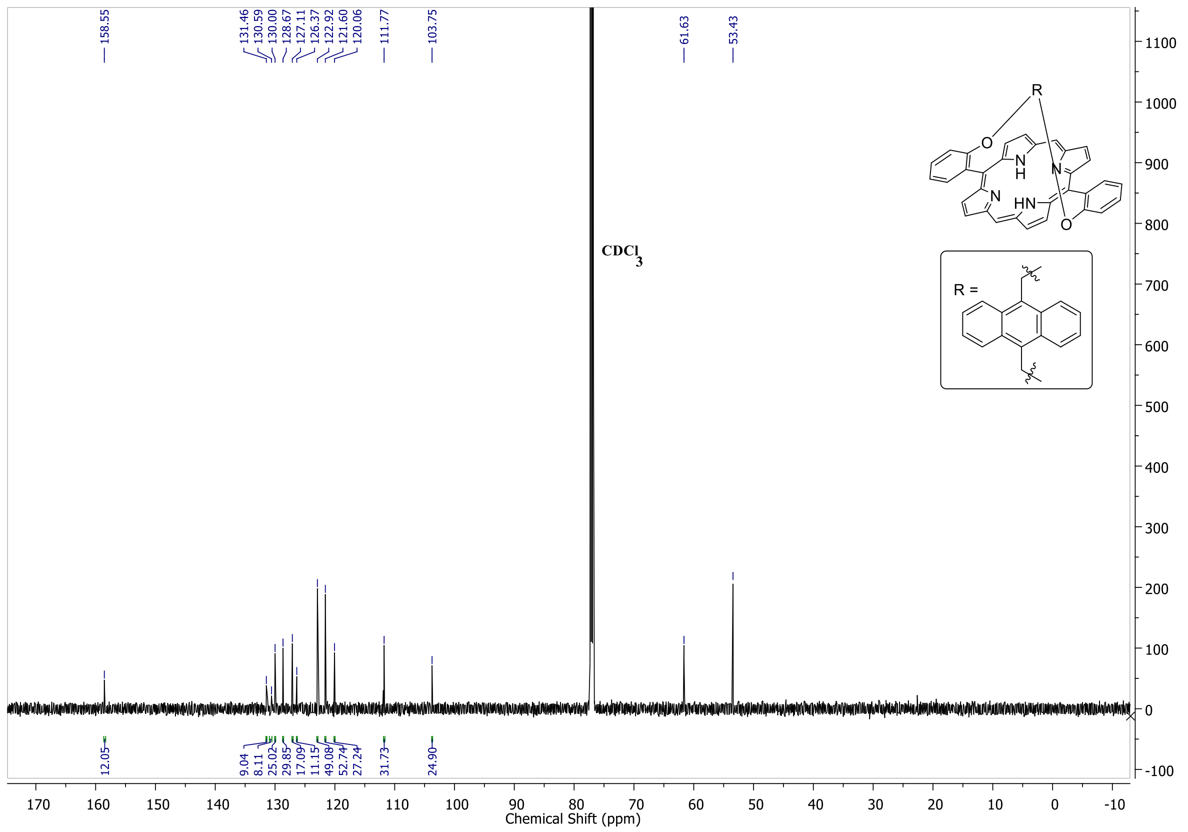

Figure S18: ^{13}C NMR spectrum of porphyrin **19** in $CDCl_3$.

2.2 NMR spectra of porphyrin 22

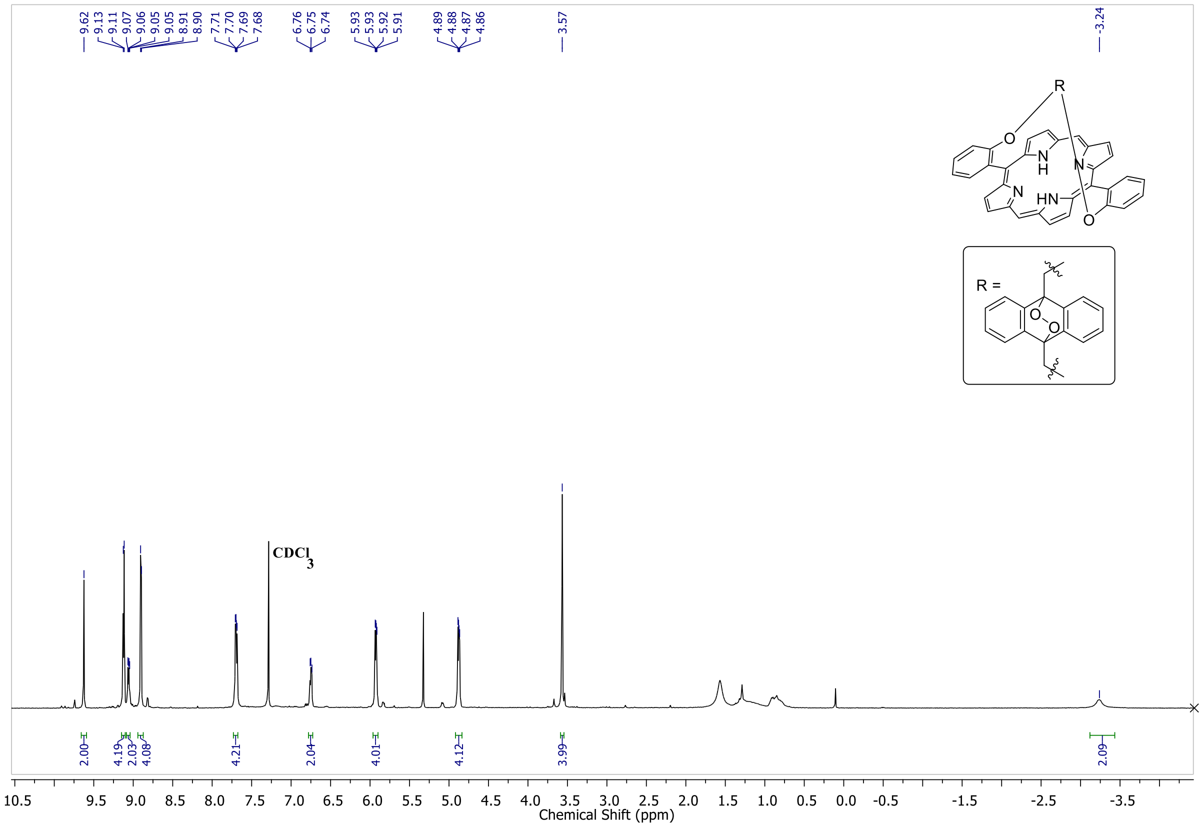

Figure S19: ^{1}H NMR spectrum of porphyrin **22** in $CDCl_3$.

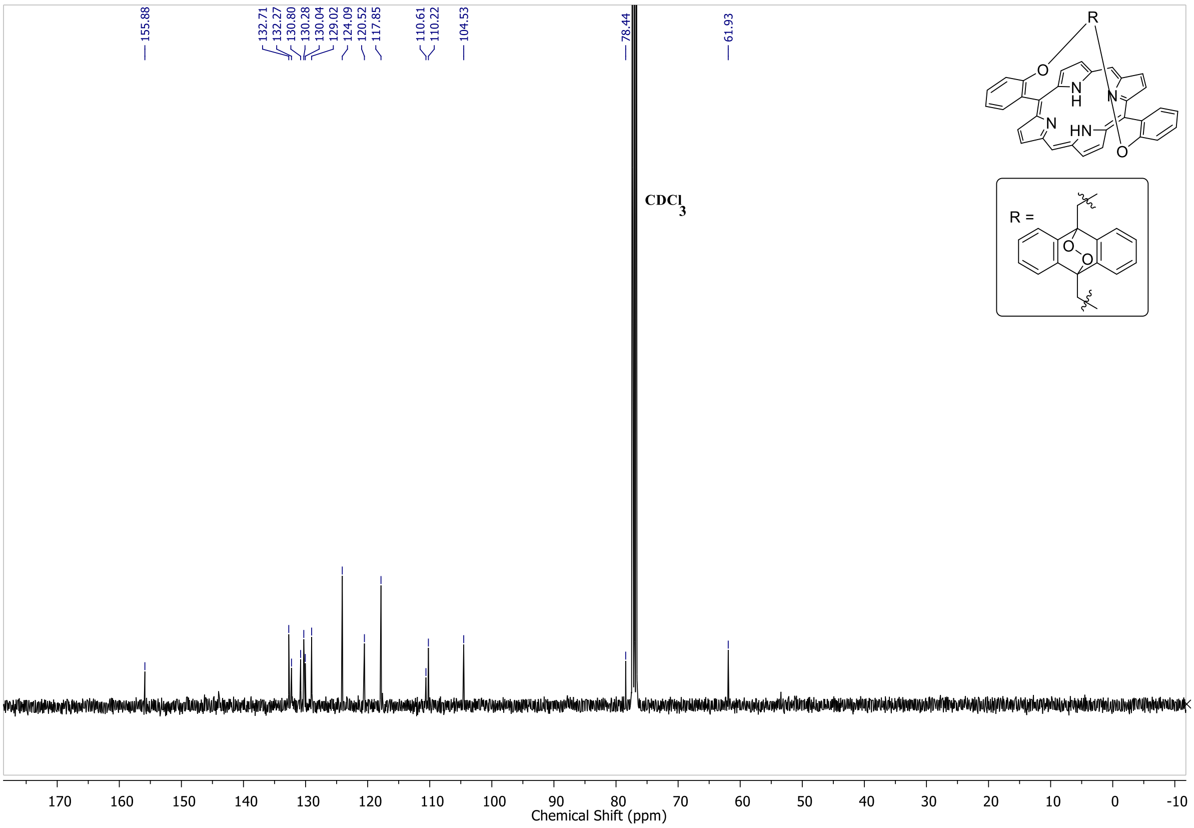

Figure S20: ^{13}C NMR spectrum of porphyrin **22** in $CDCl_3$.

2.3 NMR spectra of porphyrin 16

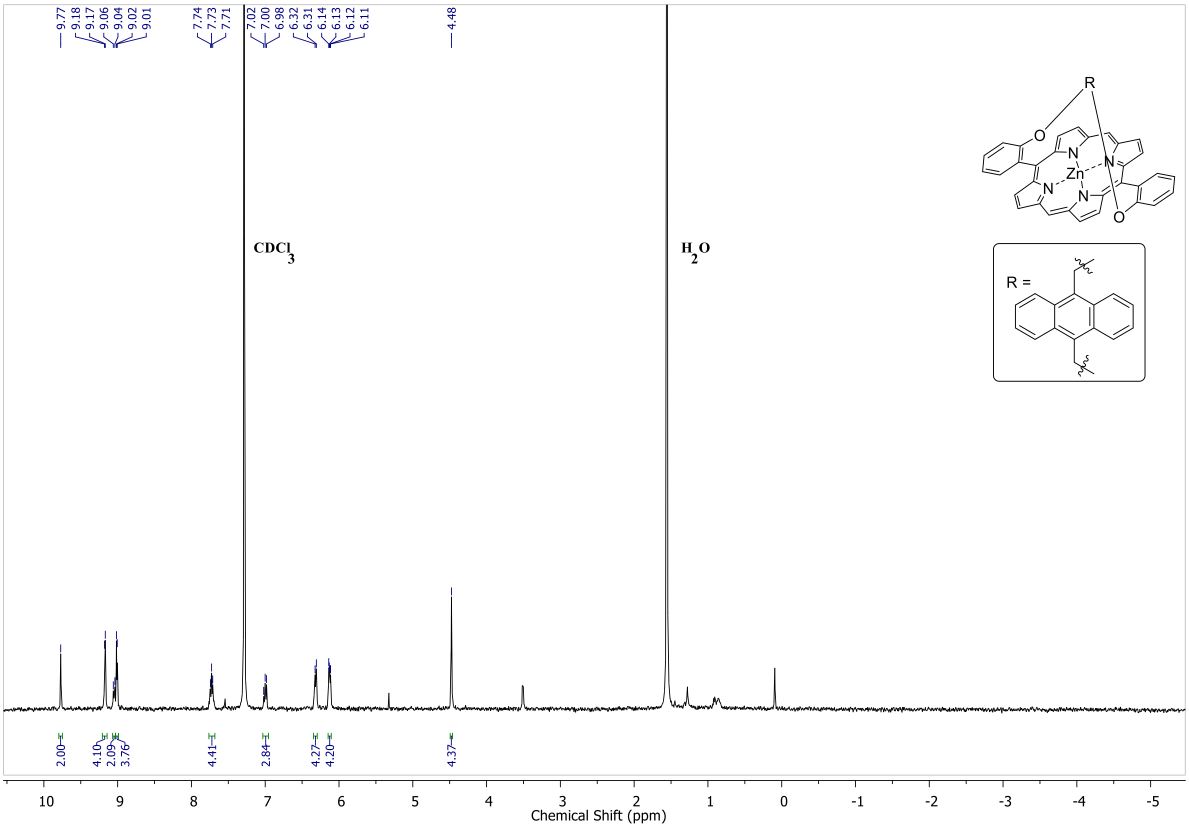

Figure S21: ^{1}H NMR spectrum of porphyrin **16** in $CDCl_3$.

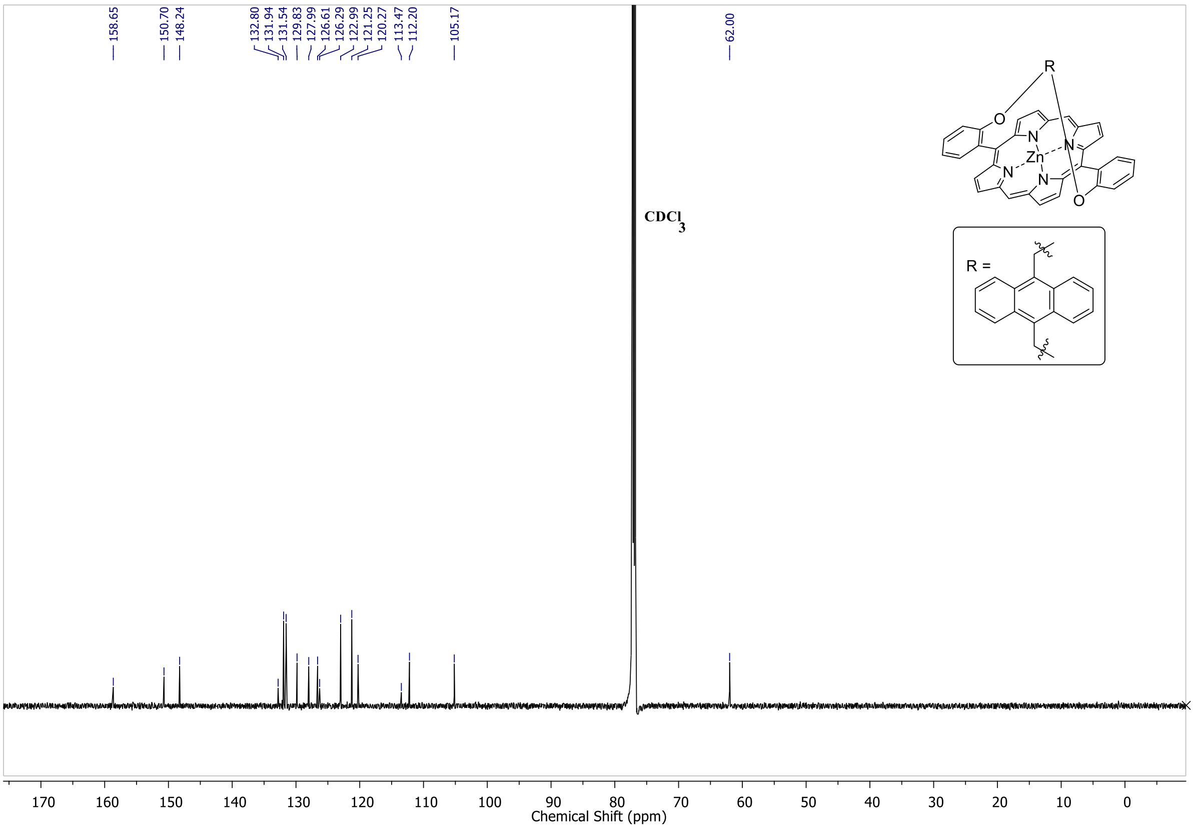

Figure S22: ^{13}C NMR spectrum of porphyrin **16** in $CDCl_3$.

2.4 NMR spectra of porphyrin 20

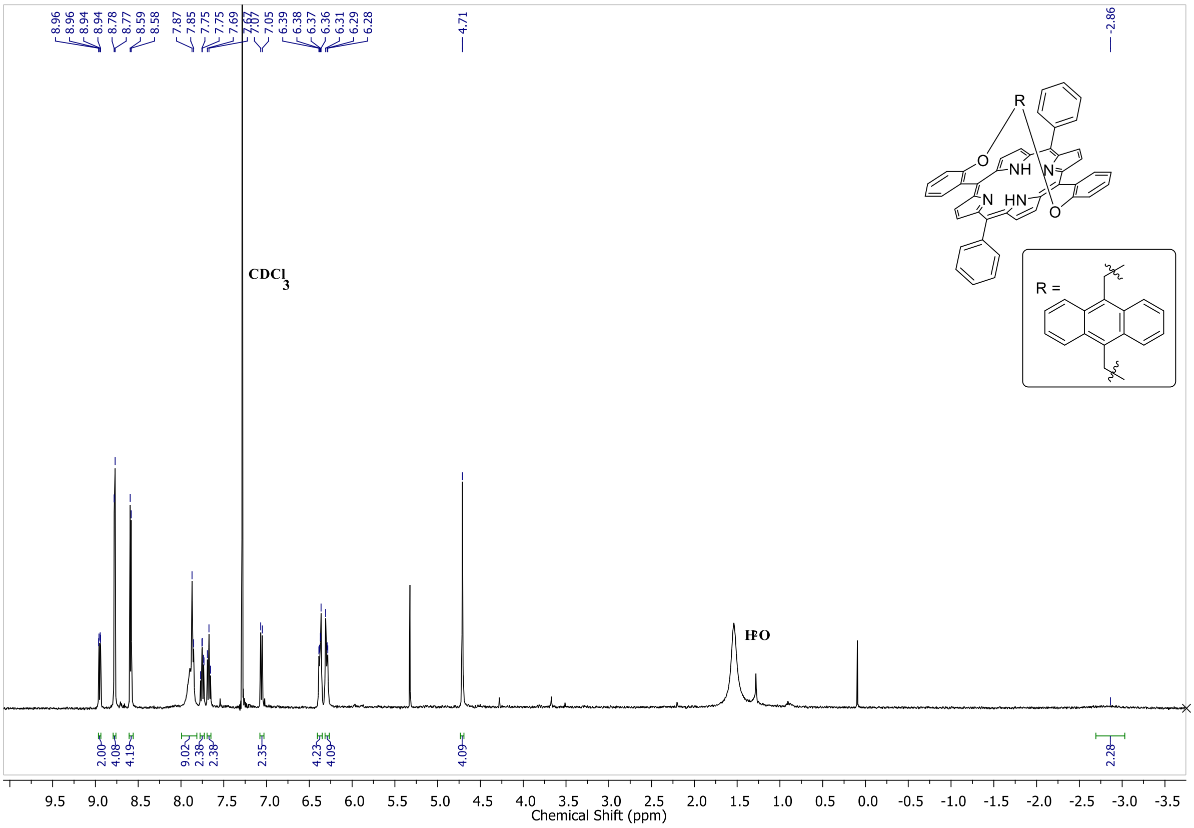

Figure S23: ^{1}H NMR spectrum of porphyrin **20** in $CDCl_3$.

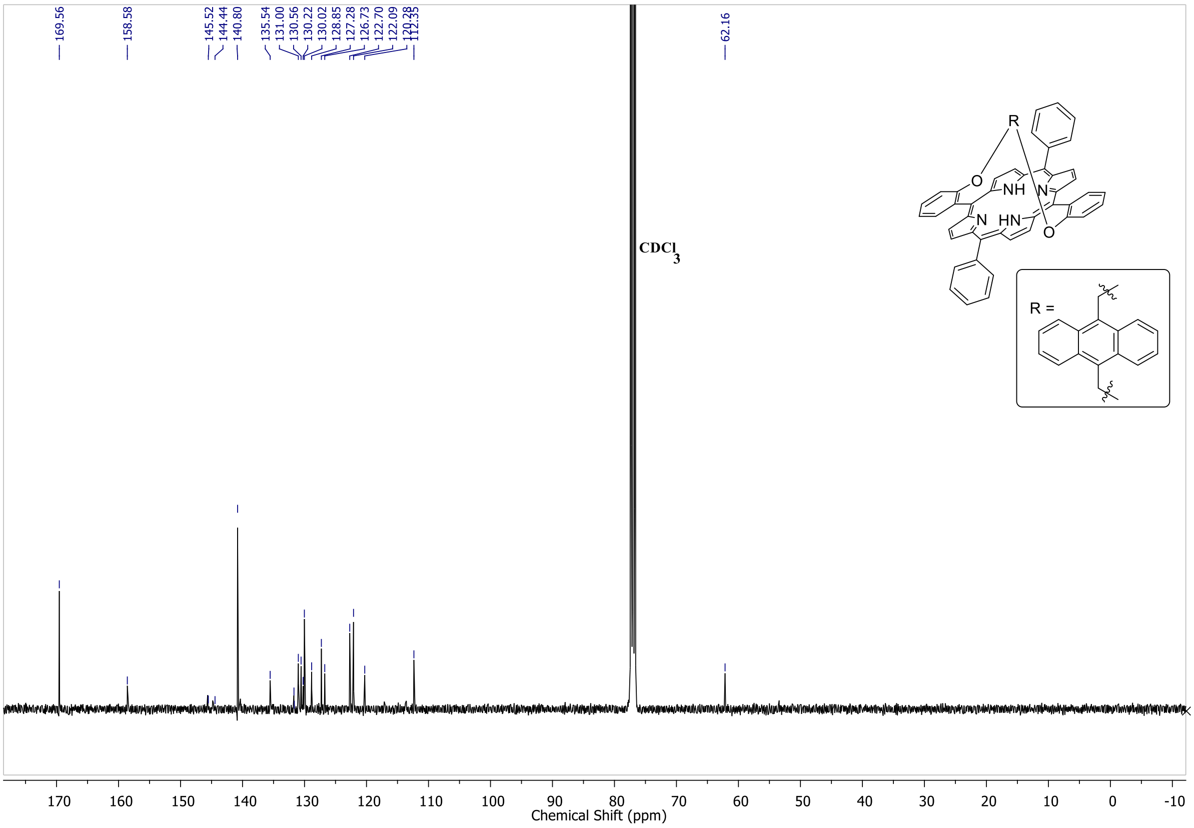

Figure S24: ^{13}C NMR spectrum of porphyrin **20** in $CDCl_3$.

2.5 NMR spectra of porphyrin 23

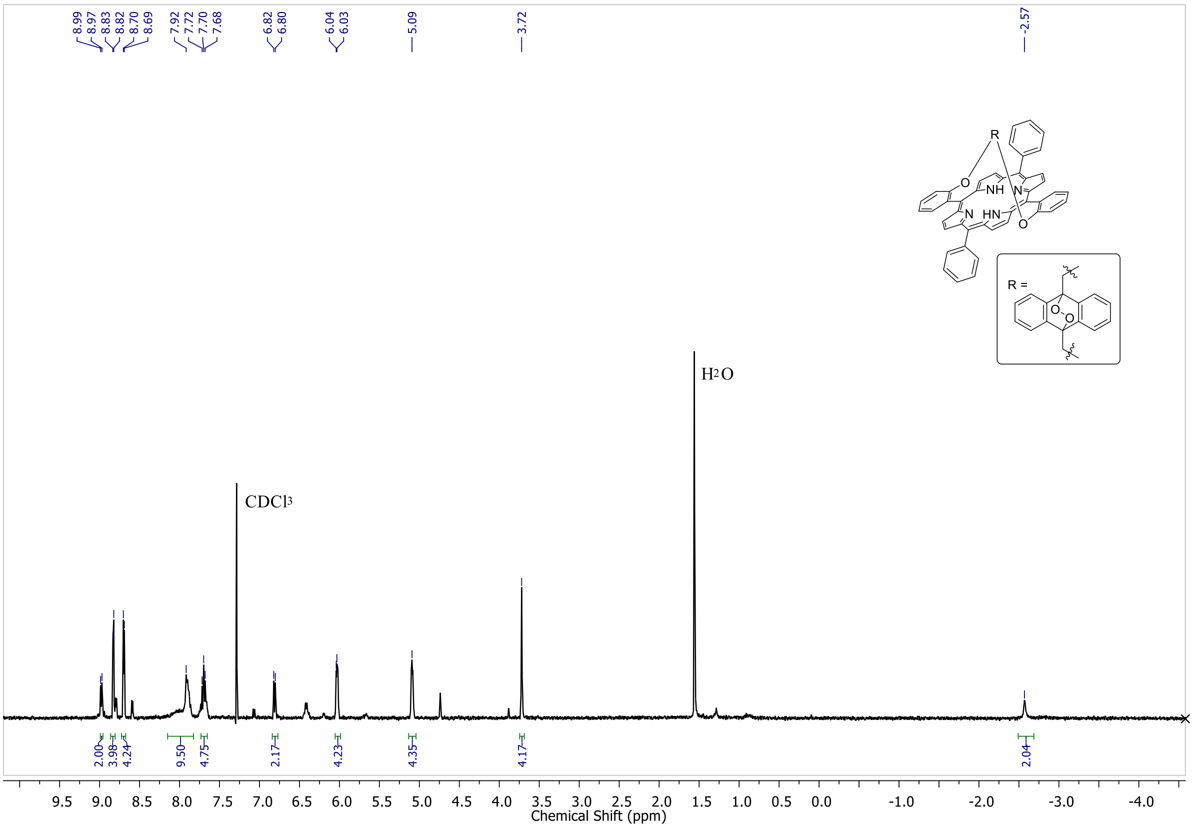

Figure S25: ^{1}H NMR spectrum of porphyrin **23** in $CDCl_3$.

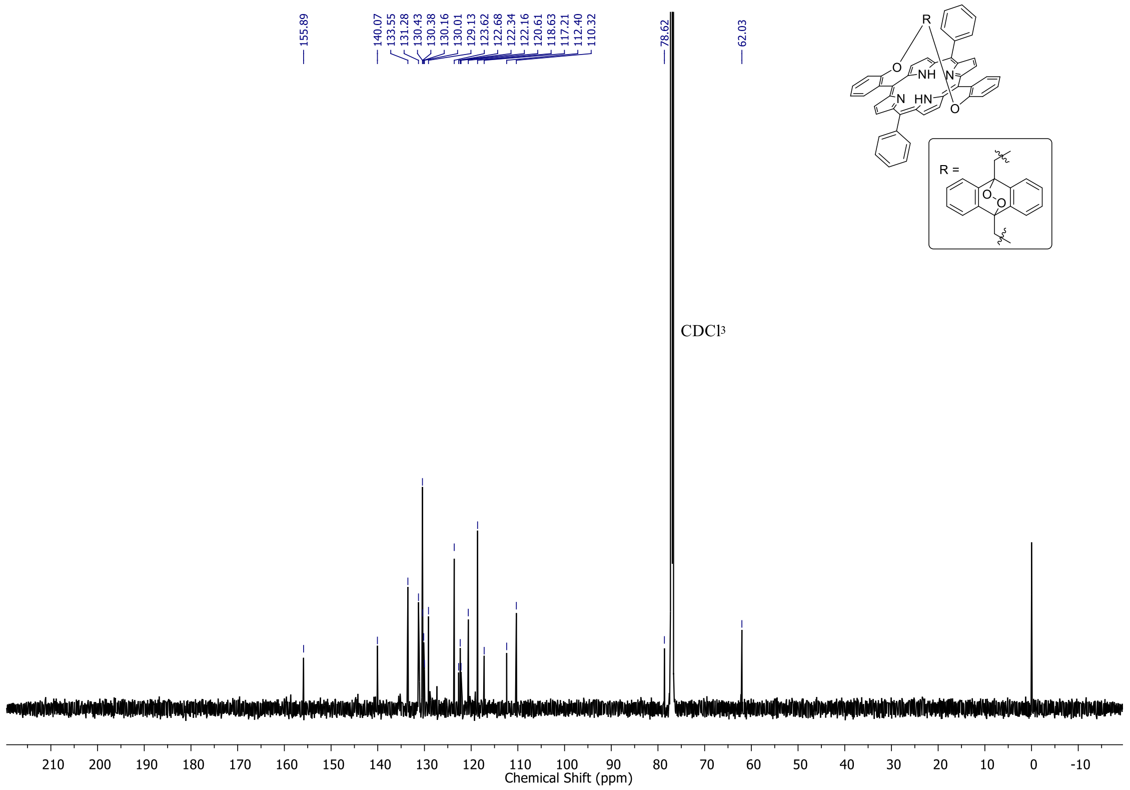

Figure S26: ^{13}C NMR spectrum of porphyrin **23** in $CDCl_3$.

2.6 NMR spectra of porphyrin 17

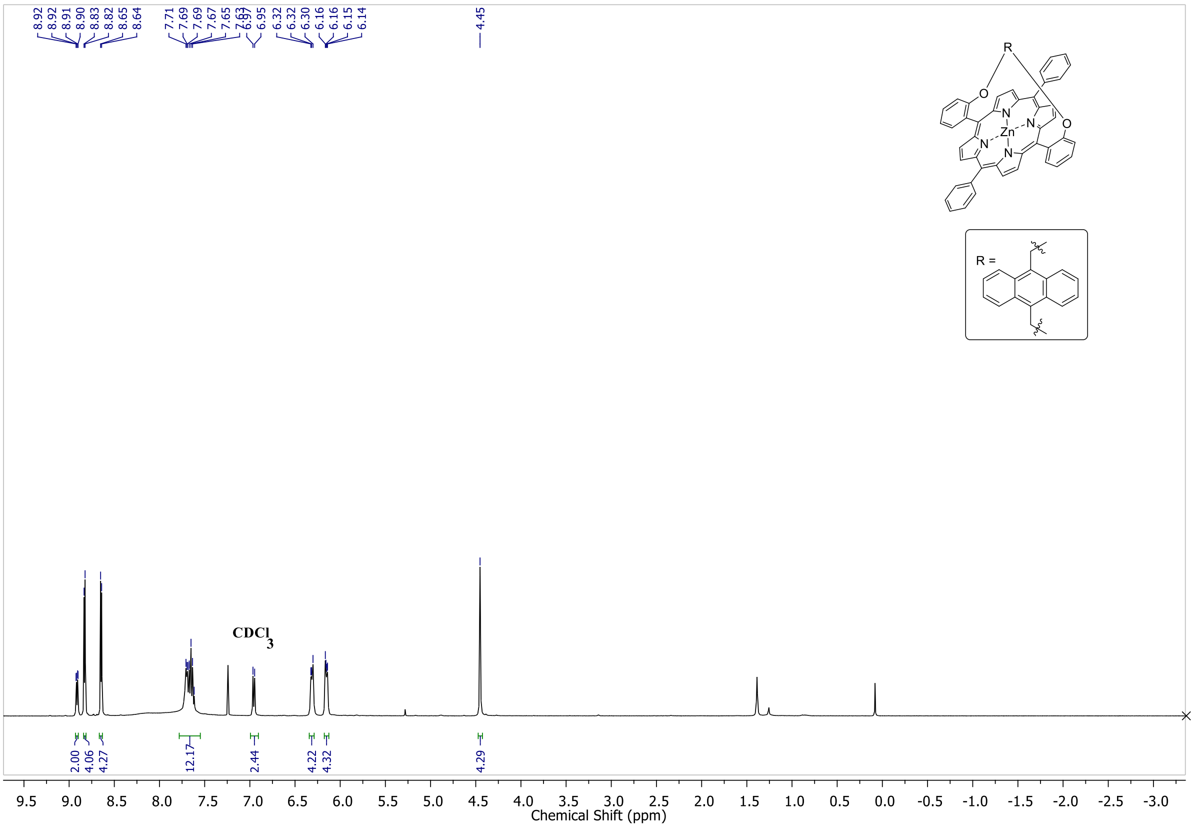

Figure S27: ^{1}H NMR spectrum of porphyrin **17** in $CDCl_3$.

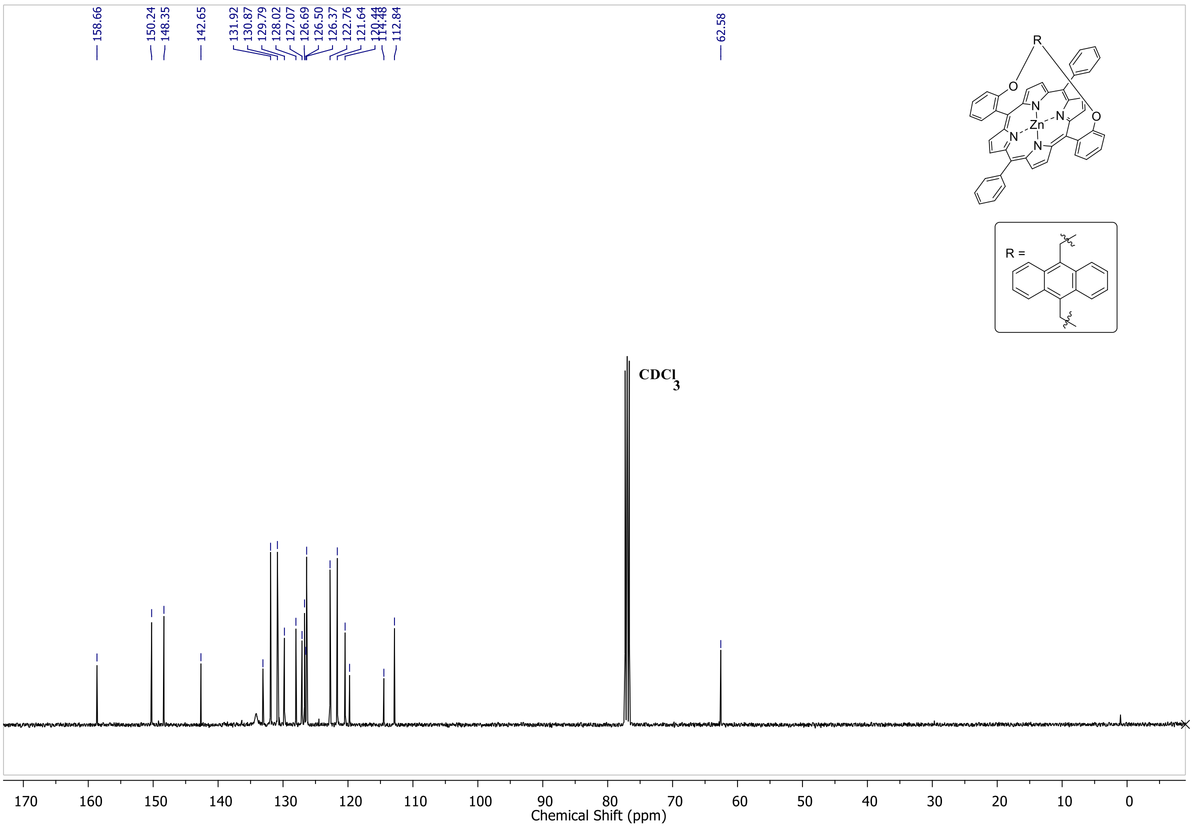

Figure S28: [13] C NMR spectrum of porphyrin **17** in $CDCl_3$.

2.7 NMR spectra of porphyrin 21

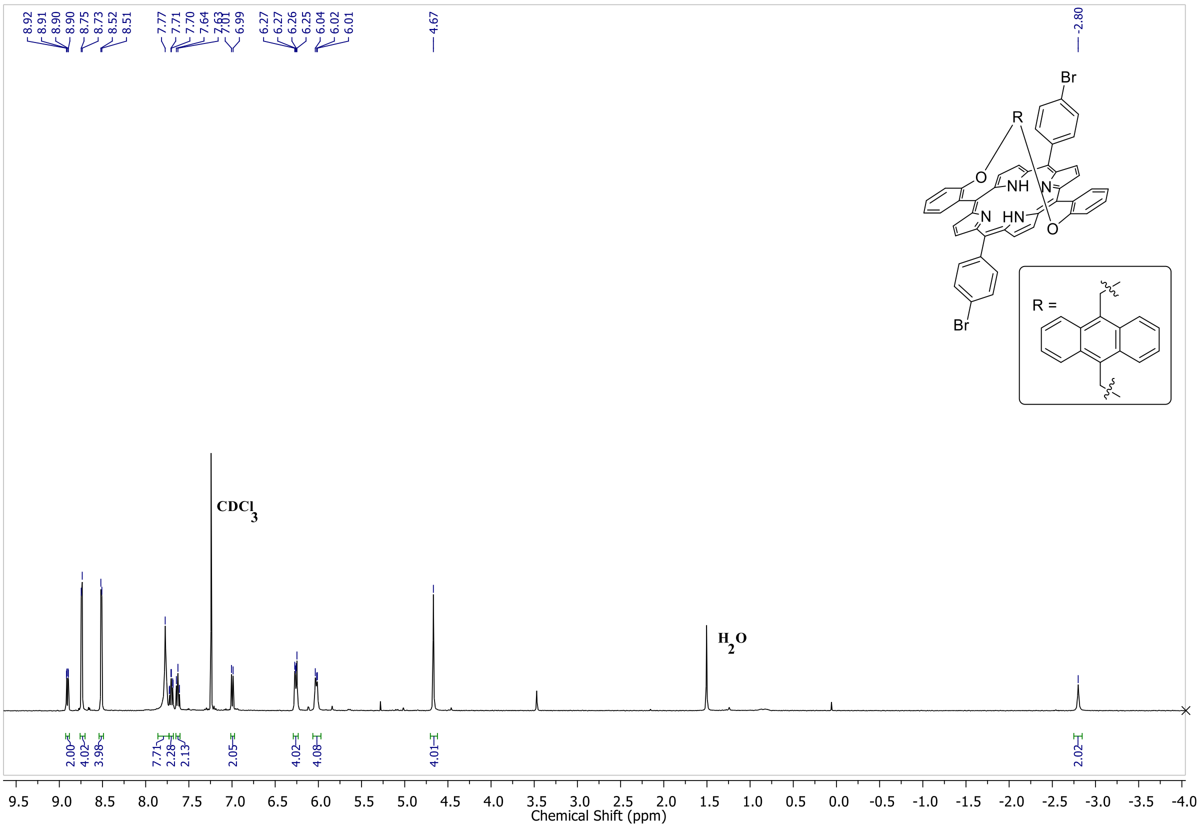

Figure S29: ^{1}H NMR spectrum of porphyrin **21** in $CDCl_3$.

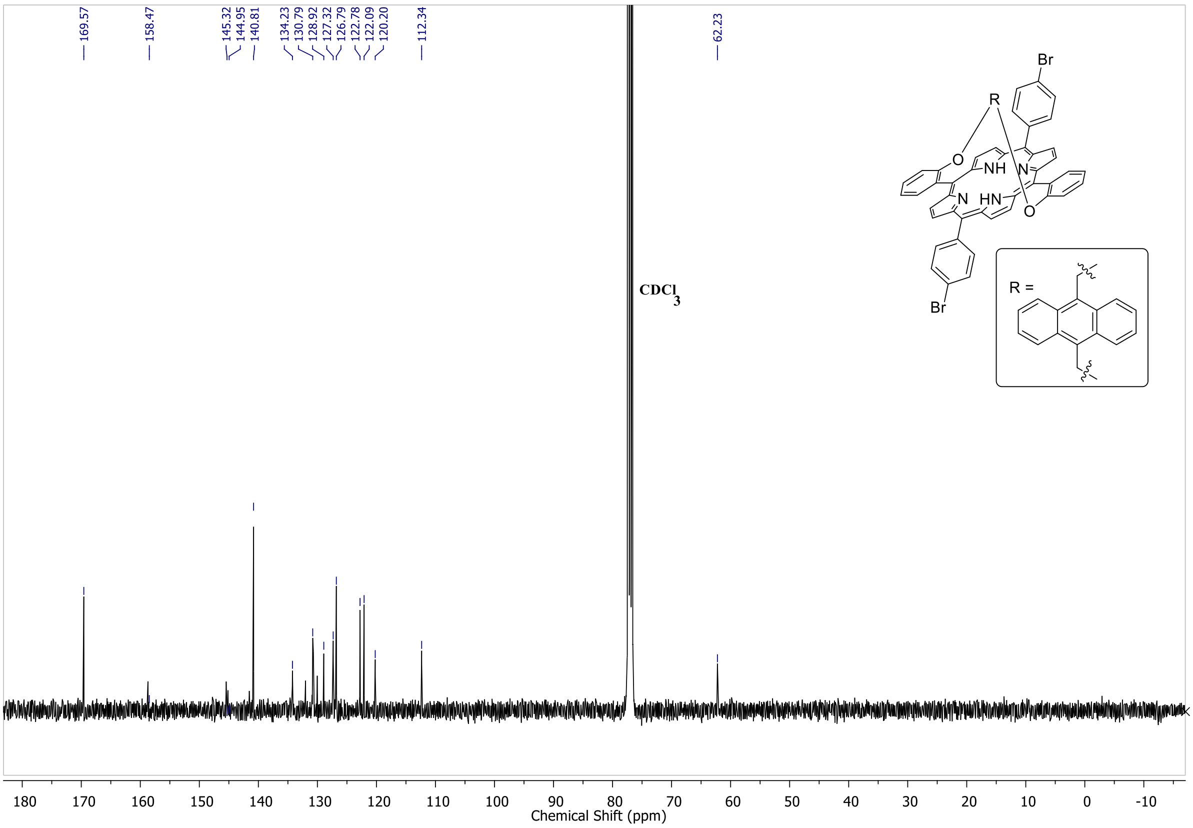

Figure S30: ^{13}C NMR spectrum of porphyrin **21** in $CDCl_3$.

2.8 NMR spectra of porphyrin 24

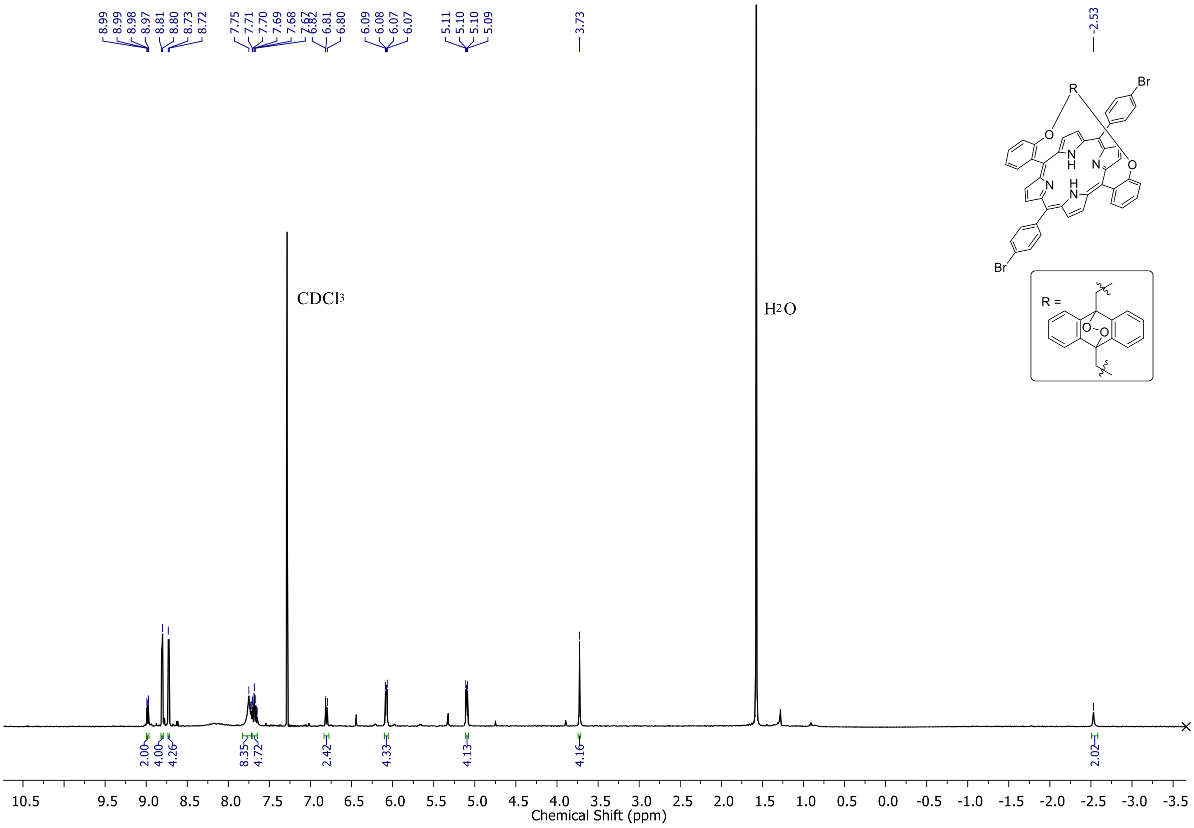

Figure S31: ^{1}H NMR spectrum of porphyrin **24** in $CDCl_3$.

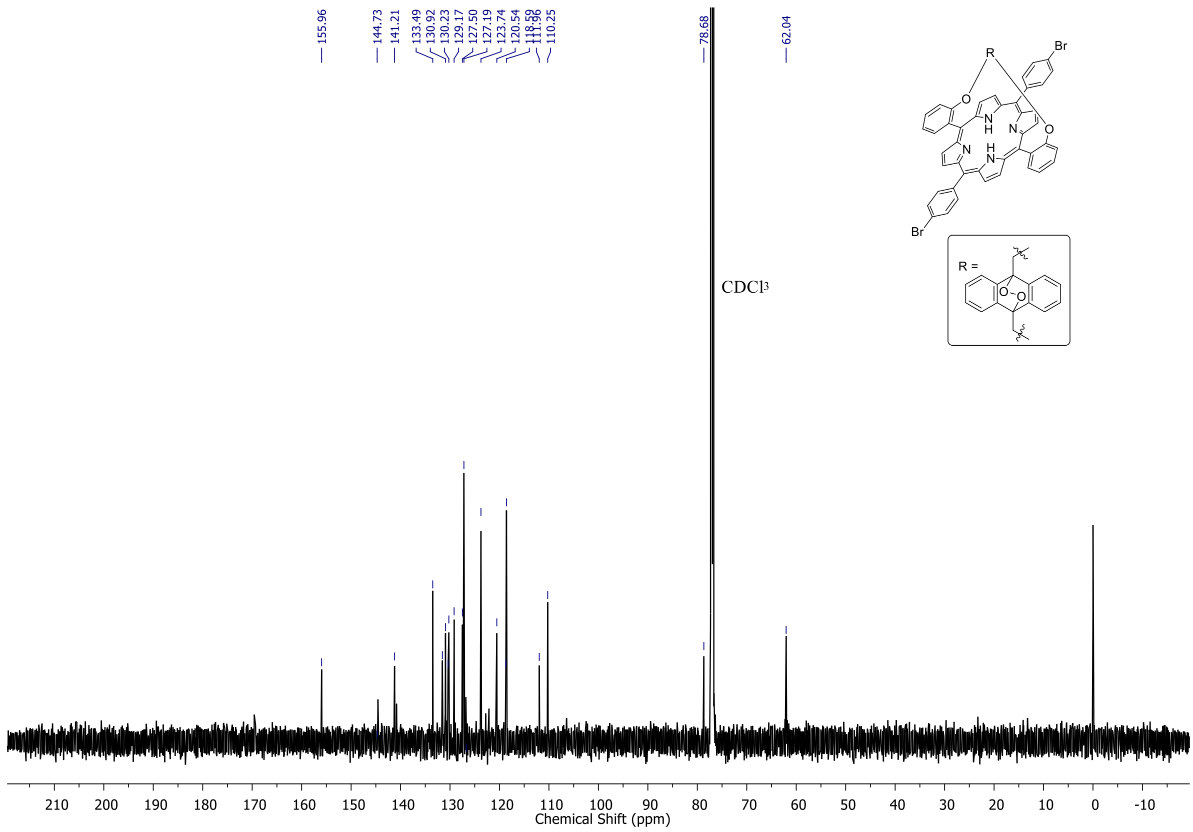

Figure S32: ^{13}C NMR spectrum of porphyrin **24** in $CDCl_3$.

2.9 NMR spectra of porphyrin 18

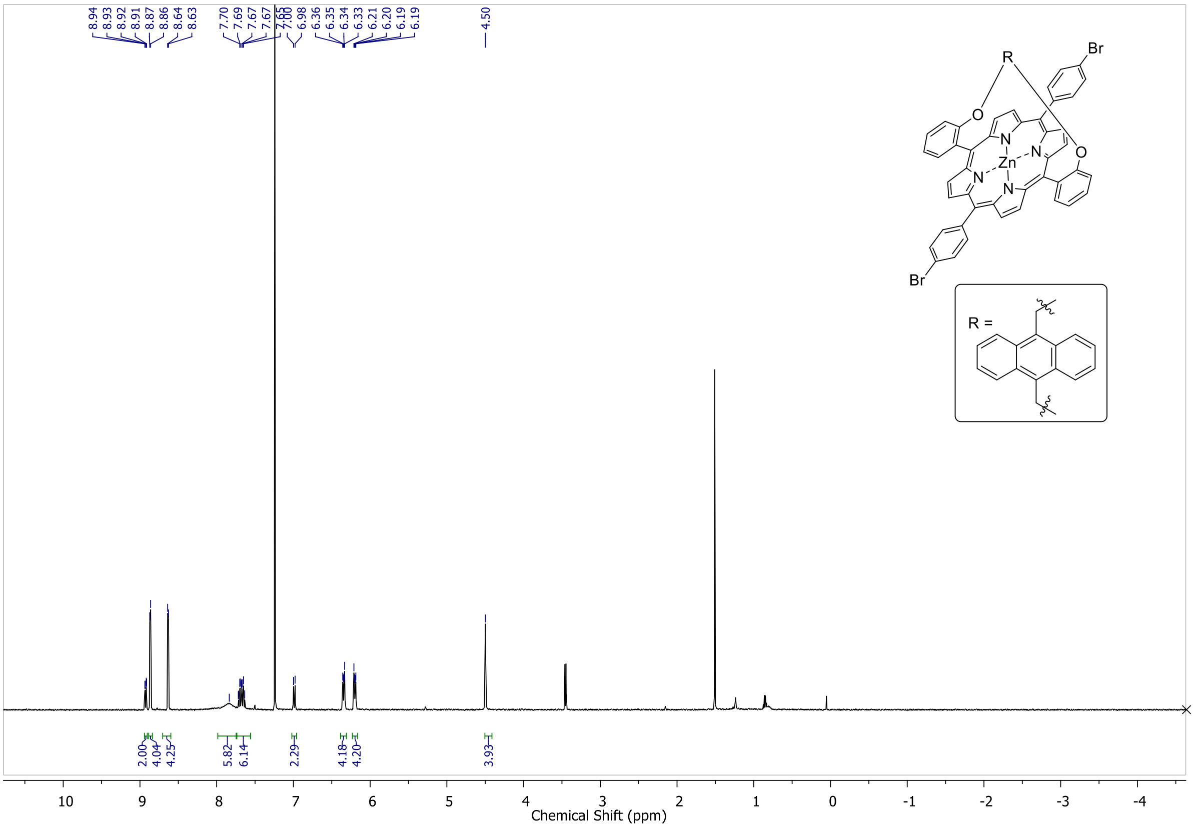

Figure S33: ^{1}H NMR spectrum of porphyrin **18** in $CDCl_3$.

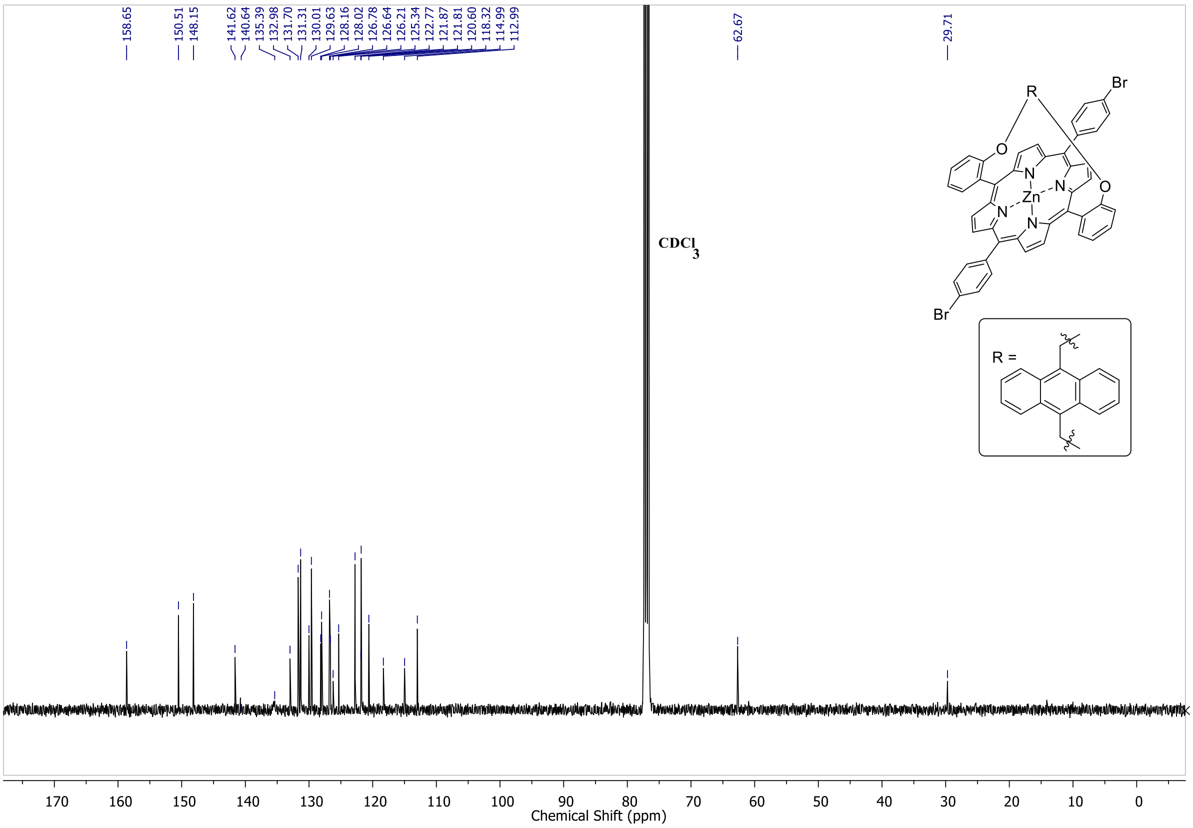

Figure S34: ^{13}C NMR spectrum of porphyrin **18** in $CDCl_3$.
